# Establishment of Gut Microbiome During Early Life and Its Relationship With Growth in Endangered Crested Ibis (*Nipponia nippon*)

**DOI:** 10.3389/fmicb.2021.723682

**Published:** 2021-08-09

**Authors:** Ying Zhu, Yudong Li, Haiqiong Yang, Ke He, Keyi Tang

**Affiliations:** ^1^Institute of Qinghai-Tibetan Plateau, Southwest Minzu University, Chengdu, China; ^2^Sichuan Province Laboratory for Natural Resources Protection and Sustainable Utilization, Sichuan Provincial Academy of Natural Resource Sciences, Chengdu, China; ^3^Emei Breeding Center for Crested Ibis, Emei, Chengdu, China; ^4^College of Animal Sciences and Technology, Zhejiang A&F University, Hangzhou, China; ^5^College of Life Sciences, Sichuan Normal University, Chengdu, China

**Keywords:** crested ibis (*Nipponia nippon*), host growth, gut microbiota diversity, non-linear change, microbial recruitment patterns, diet change, environment change

## Abstract

Gut microbiota during early life could influence host fitness in vertebrates. Studies on how gut microbiota colonize the gut in birds using frequent sampling during early developmental stages and how shifts in microbiota diversity influence host growth are lacking. Here, we examine the microbiome profiles of 151 fecal samples from 14 young crested ibis (*Nipponia nippon*), an endangered bird species, collected longitudinally across 13 time points during the early stages of development and investigated their correlation with host growth. Gut diversity showed a non-linear change during development, which involved multiple colonization and extinction events, mainly associated with Proteobacteria and Firmicutes. Gut microbiota in young crested ibis became more similar with increasing age. In addition, gut microbiota exhibited a strong temporal structure and two specific developmental stages; the beginning of the latter stage coincided with the introduction of fresh loach, with a considerable increase in the relative abundance of Fusobacteria and several Firmicutes, which may be involved in lipid metabolism. Crested ibis chick growth rate was negatively correlated with gut microbiota diversity and negatively associated with the abundance of Halomonadaceae, Streptococci, Corynebacteriaceae, and Dietziaceae. Our findings highlight the importance of frequent sampling when studying microbiome development during early stages of development of vertebrates. The role of microbial diversity in host growth during the early stages of development of birds warrants further investigations.

## Introduction

Gut microbiota participate in host health maintenance, nutrition uptake, digestion, energy release, detoxification, gut development, and the regulation of host physiology and immunity (Waite and Taylor, 2015; Grond et al., 2018), and hence disruptions of normal microbiome could lead to loss of such benefits (Reid et al., 2011; Lewis et al., 2015). In addition, gut microbial communities could influence host fitness, including survival (Kohl et al., 2018), reproductive performance (Hamdi et al., 2011; Rosengaus et al., 2011), and adaption (Blumstein et al., 2017).

In birds, in addition to ecological variables such as diet (Torok et al., 2011; Huang et al., 2018), captivity status (Xenoulis et al., 2010; Wienemann et al., 2011), locality (Hird et al., 2014; Capunitan et al., 2020), and seasons (Lewis et al., 2016; Góngora et al., 2021), biological factors, such as age, are important factors shaping gut microbiota. For example, gut microbiota during early life has been reported to differ markedly from gut microbiota in conspecific adults, as observed in house sparrow (*Passer domesticus*) (Kohl et al., 2019), black-legged kittiwakes (*Rissa tridactyla*) (van Dongen et al., 2013), chinstrap penguins (*Pygoscelis antarctica*) (Barbosa et al., 2016), and folivorous hoatzin (*Opisthocomus hoazin*) (Godoy-Vitorino et al., 2010).

Gut bacterial communities are relatively stable in adults, however, during early life, they are much more transient and dynamic, based on studies conducted on non-avian vertebrates (Grond et al., 2018). Several recent studies have investigated the early establishment of gut microbiota in domestic birds such as chicken (Yin et al., 2010; Oakley et al., 2014; Ballou et al., 2016), turkey (Wilkinson et al., 2017), and wild birds, such as in house sparrows (Kohl et al., 2019), great tits (*Parus major*) (Teyssier et al., 2018), little penguins (*Eudyptula minor*), short-tailed shearwaters (*Ardenna tenuirostris*) (Dewar et al., 2017), black-legged kittiwakes (van Dongen et al., 2013), folivorous hoatzin (Godoy-Vitorino et al., 2010), chinstrap penguins (Barbosa et al., 2016), and dunlin (*Calidris alpina*) (Grond et al., 2017). Such studies on gut microbiota diversity trends with aging during early life have yielded varying results across species. For example, older nestlings have lower microbial diversity than younger nestlings, which was observed in great titis (Teyssier et al., 2018), chicken (Oakley et al., 2014; Ballou et al., 2016), and chinstrap penguins (Barbosa et al., 2016). Opposite trends were observed in black-legged kittiwakes (van Dongen et al., 2013). In addition, some studies have revealed that age does not influence gut microbiota diversity in nestlings in house sparrow (Kohl et al., 2019) and short-tailed shearwaters (Dewar et al., 2017). Furthermore, some species, such as turkey, exhibited much more complex gut microbiota diversity patterns (Wilkinson et al., 2017). These studies have reported strong fluctuations in community composition in the first stages of nestling development. The diverse results across species during early life are thought to be influenced mainly by environmental factors (e.g., chick rearing conditions) (Hird et al., 2014; Grond et al., 2018) and diet (Kohl et al., 2018). Furthermore, infrequent sampling could result in distinct conclusions with regard to gut microbiota colonization processes (de Muinck and Trosvik, 2018). Longitudinal studies with frequent sampling are required to illustrate a comprehensive overview of gut microbiota dynamics in birds (Grond et al., 2018).

Microbial diversity could influence host fitness through its effects on chick growth rate, which is considered a strong predictor of survival later in life (Magrath, 1991). It is unclear whether an increase in diversity could enhance or limit host growth based on the results of the relatively few studies available currently. Most studies have showed that microbial diversity limits growth based on direct evidence come from the comparison of germ-free chickens and conventional chicks, where germ-free chicks grow more rapidly than conventional chicks (Forbes and Park, 1959). Furthermore, antibiotic treatment (assuming antibiotics decrease microbiota diversity) increased growth in chicks (Potti et al., 2002; Dibner and Richards, 2005; Kohl et al., 2018), potentially via the enhancement of food conversion efficiency (Kohl et al., 2018). However, studies performed on ostrich (*Struthio camelus*) have revealed contradicting results at different stages of development: gut microbial diversity was strongly positively associated with growth only during the first week after hatching, and microbial diversity was negatively related with growth after the first week (Videvall et al., 2019). The limited studies and inconsistent results on the association between microbial diversity and host growth in birds highlight the need for further research into the role of microbial diversity in host growth during the early lives of birds.

Crested ibis (*Nipponia nippon*) is an endangered bird species currently found in China, Japan, and South Korea. *Ex situ* conservation, which aims to reintroduce captive individuals to the wild, has been established in China since 1981 (Ding, 2004), where crested ibis are raised in controlled conditions (indoor) after hatching and moved outdoors until reaching approximately 44 days or age. The chicks are raised separately from their parents and are mainly fed on loaches. During the indoor stage, crested ibis undergo a diet change and two environmental changes (Ding, 2004). Gut microbiota composition at hatching is distinguished from other life stages (Ran et al., 2021), however, it is unclear whether community structure is mainly influenced by biological (such as age) or ecological variables (such as diet and environmental changes) during early life. In addition, variation in chick growth rate has been previously observed but whether it is due to gut microbiota remains to be determined.

In the present study, we describe gut microbiota diversity of crested ibis using 16S rRNA amplicon sequencing using frequent sampling strategy. Repeated fecal sampling was conducted under controlled conditions from at hatch until day 44, which corresponds to the developmental stages in this species (Ding, 2004). Our study aims to (1) analyze gut microbiota diversity trends and demonstrate microbial recruitment patterns and colonization processes during growth. (2) Investigate the influence of two ecological variables (diet and environment) and three biological variables (age, sex, and genetic relatedness) on gut microbiota structure and elucidate the functions of the key bacterial groups. (3) Demonstrate the effect of gut microbiota diversity and composition on growth.

## Materials and Methods

### Sample Collection

**FIGURE 1:**
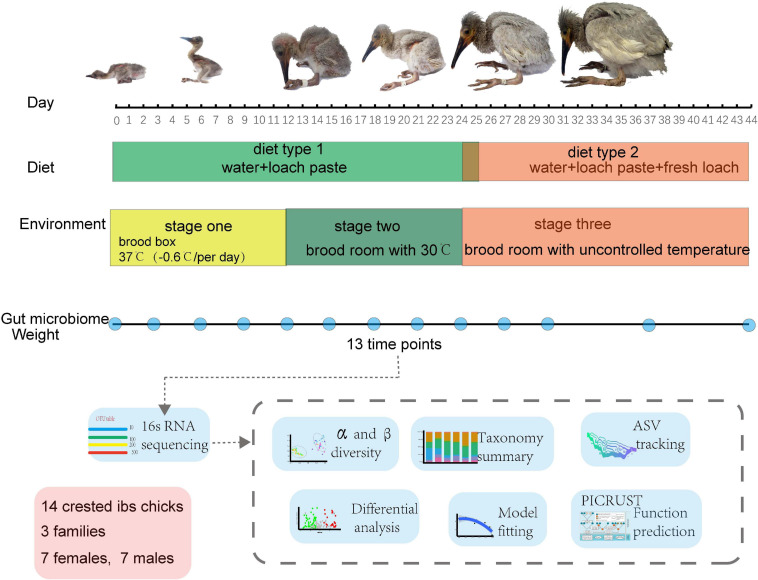
Overview of the study design and sample collection.

Fourteen newborn crested ibis from three families were raised at the Emei breeding center of crested ibis and housed in separate incubators (Supplementary Table 1). Crested ibis chick were housed in the brood box with the temperature −0.6°C per day from 37°C, fed on loach paste at hatching until day 12 (stage one and diet type 1 in Figure 1), and moved to the brood room with consistent temperature of 30°C and where they were fed loach paste until day ∼22 to ∼25 (stage two and diet type 1 in Figure 1). Finally, the birds lived in the brood room without controlled temperature, and fresh loach was added to their formula (stage three and diet type 2 in Figure 1). The addition of fresh loach and controlled temperature were both to adapt chick development (Ding, 2004). None of the crested ibis were administered antimicrobial drugs during the sampling period. Fresh feces (*n* = 182) were obtained from crested ibis, from hatching to day 44 between May and June 2018 (repeatedly sampling at hatching and days 3, 6, 9, 12, 15, 18, 21, 24, 27, 30, 37, and 44; Figure 1 and Supplementary Table 1). Crested ibis were weighed every day before the first feeding (Supplementary Table 1). Feces were collected using sterile spoons and placed in sterile tubes. The fecal samples were stored in liquid nitrogen until DNA extraction. Sex was identified using the CD1 gene (He et al., 2013).

### DNA Extraction, 16S rRNA Gene Sequencing, and Data Processing

DNA extraction from fecal samples was performed using the hexadecyltrimethylammonium bromide method. The V3–V4 regions of the 16S rRNA genes were amplified using primers 341F: 5′-CCTAYGGGRBGCASCAG-3′ and 806R: 5′-GGACTACNNGGGTATCTAAT-3′. Samples with failed PCR amplification were abandoned (*n* = 31), resulting in 151 samples in the subsequent process. Paired-end sequencing (2 × 250 PE) was conducted at the Nova sequencing company (Novaseq, Tianjin, China) on an Illumina Novaseq 6000 sequencing system (Illumina, San Diego, CA, United States).

Processing of sequence data was conducted using a combination of usearch v10.0.240 and vsearch v2.15.0 (Liu et al., 2021). Dereplication was conducted using the “derep_fulllength” function in vsearch with a minimum unique size of 10, to eliminate artefactual reads. The Divisive Amplicon Denoising Algorithm was performed using the unoise3 function in usearch for correcting amplicon errors (Callahan et al., 2016), yielding 1404 amplicon sequence variations (ASVs). We obtained a total of 9,693,246 high-quality reads from 151 samples (averaging 64193.7 and ranging from 5,0136 to 6,9673 reads per sample). We observed 1404 ASVs after denoising. ASV feature tables were created with the “usearch_global” function in vsearch with a similarity probability of 0.97. Taxonomic profiling was done against the Ribosomal Database (rdp_16s_v16_sp)^1^ using the sintax function in vsearch. Sequencing library sizes were normalized to 50,000 to adjust for sample differences.

### Statistical Analysis

All statistical tests were conducted in R (version 4.0.2, 2020-06-22). Sample differences in sequencing library size were normalized to 50,000 using the “Vegan” package (Oksanen et al., 2020). Shannon index were calculated in R package “Vegan”. Bray–Curtis distances and weighted UniFrac distances (Lozupone and Knight, 2005) were computed with the “beta_div” function in usearch.

Permutational multivariate analysis of variance (PERMANOVA) was conducted to detect the effects of age, sex, family group (genetic relatedness), individuality, temperature, and diet using the “adonis” function in the “Vegan” package using both Bray–Curtis distances and weighted UniFrac distances with 999 permutations (Oksanen et al., 2020). Principal co-ordinates analysis (PcoA) of Bray–Curtis distance matrices was conducted using the “cmdscale” function.

We used a polynomial linear mixed-effects model to estimate smooth terms in order to fit non-linearity among the Shannon index, Bray–Curtis distance, and age as the predictor variable, with stage used as the covariate factor and individual ID controlled. The relative abundances of taxa of interest were modeled to age, with stage as a covariate factor and individual ID controlled in the linear mixed-effects model.

We modeled young bird growth (weight change per day between time *t* and *t* + 1) to microbial diversity at time t, including age at time *t* and diet type at time *t*, as the covariate and individual ID as a random factor (Videvall et al., 2019) using linear mixed-effect models. To investigate the effect of specific microbial phyla, classes, and families on growth, we also modeled the growth to relative abundances of specific taxa (details in Supplementary Data 1). All the linear mixed-effects models were conducted in the NLME package in R (Pinheiro et al., 2021).

The bacterial metagenome was predicted from the 16S rRNA database (Greengenes Database, version gg_13_5) and functional profiles were inferred from the Kyoto Encyclopedia of Gene and Genomes (KEGG) using PICRUSt (phylogenetic investigation of communities by reconstruction of unobserved states) (Douglas et al., 2018).

Differential abundances between two adjacent time points and between two diet types (for microbiota composition and functional profile) were detected in edgeR using a negative binomial generalized linear model with individual ID controlled (Robinson et al., 2010). The model considers sample library size and the dispersion of each ASVs or taxon, which are achieved using the calcNormFactors function. ASVs or taxa with the mean relative abundances ≥0.01% were retained. The *P* values were adjusted for multiple tests with the Benjamini and Hochberg false discovery rate of 0.05 (Benjamini and Hochberg, 1995). ASVs or taxa were significantly abundant or depleted if they had a corrected *P* value < 0.05 and | logFC| > 2.0 (details in Supplementary Data 2).

Spearman’s rank correlation coefficients were used to examine the associations between specific genera and pathways (both significantly related to diet) and calculated in the Hmisc package in R (Harrell, 2021).

Sankey plots were produced in imageGP^2^ by tracking ASVs with mean relative abundances across all samples greater than 0.1%. Heatmaps were illustrated using the pheatmap package in R (Kolde and Kolde, 2015). Other data visualization procedures were performed using the ggplot2 package (Wickham, 2011).

For the linear mixed-effects models, we removed variables that neither affected the dependent variable nor contributed to the model in order to the keep the model simple. Additionally, we removed variables that could potentially induce serious multicollinearity and reduce the precision of the estimated coefficients.

## Results

### Gut Microbiota Varied Over Time

**FIGURE 2:**
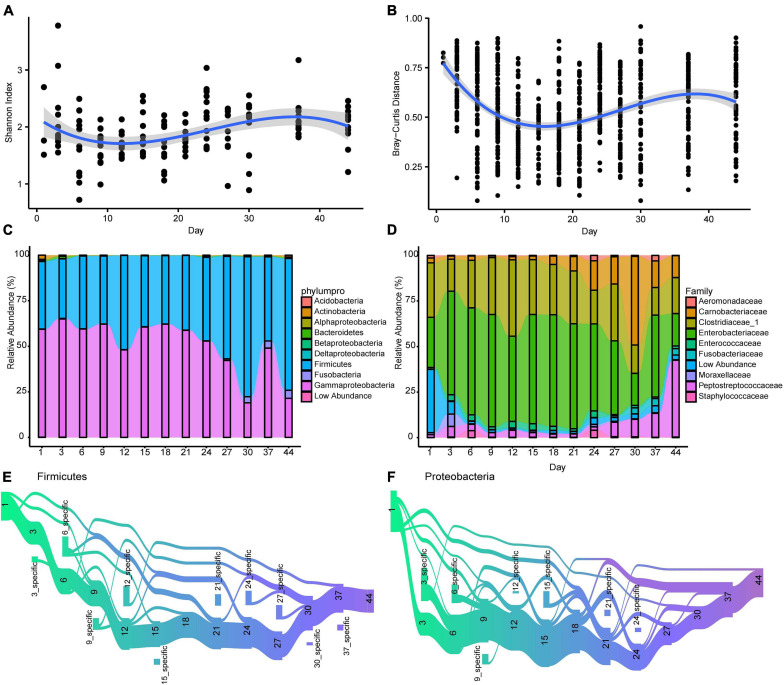
The longitudinal dynamic of gut microbiota in Crested ibis. **(A)** The alpha diversity of gut microbiota over time. The shadow around the lines reveals 95% CI. The alpha diversity was estimated using the Shannon index. **(B)** The beta diversity of gut microbiota over time. The Bray-Curtis distance calculated at the ASV level across microbiota from samples collected at the same time point. Higher values denoted more dissimilar gut microbiota. Samples difference in sequencing library size were normalized to 50,000 reads. **(C)** The temporal changes of bacterial relative abundance at the phylum/class level (top 10). **(D)** The temporal changes of bacterial relative abundance at the family level (top 10). **(E,F)** ASVs that the mean relative abundance across all samples larger than 0.1% are tracked using Sankey plots for both Firmicutes and Proteobacteria. The heights of the rectangles show the relative number of ASVs, and each time point has a distinct color. The lines represent the transfer of ASVs among different time points.

Gut microbiota diversity fluctuated notably over time during the first 44 days, decreasing from day 1 after birth to day 12 and then increasing until day 37 (Figure 2A). Like alpha diversity, dissimilarity within samples revealed a temporal pattern (Figure 2B). A wave trough appeared at day 15, where Bray-Curtis dissimilarity within age group was the smallest. Second peaks emerged for both Shannon diversity and Bray-Curtis distance at day 37. The temporal patterns of alpha diversity and beta diversity both fitted the polynomial age term (linear mixed-effects model; Supplementary Table 2).

With an increase in age, gut microbial community similarity within age groups increased to levels greater than in other age groups (days 12, 15, 18, 21, 24, 27, 30, 37, and 44, Supplementary Figure 1). The dissimilarity between day 44 (the oldest age we studied) and other time points revealed an overall decrease in dissimilarity with growth (Supplementary Figure 1), indicating that gut microbiota structure gradually stabilized after birth.

To identify the main ASV associated with the diversity trend, we calculated the correlation between the relative abundance of the 20 most abundant ASVs (91.7% of total abundance) and the corresponding Bray-Curtis distance. We observed that eight ASVs (ASV10∼12, ASV15∼19, 5 Proteobacteria, 2 Firmicutes, and 1 Fusobacteria, Supplementary Figure 2) were significantly correlated with Bray–Curtis distances (Supplementary Table 3).

The most prevalent phyla at different ages were Firmicutes and Proteobacteria (Gammaproteobacteria; Figure 2C and Supplementary Figure 3). The Proteobacteria and Firmicutes phyla were dominated by Enterobacteriaceae, Clostridiaceae_1, Carnobacteriaceae, and Peptostreptococcaceae, respectively, (Figure 2D and Supplementary Figure 4). Although age did not significantly influence the relative abundance of Firmicutes and Gammaproteobacteria (Firmicutes, β ± SE = 0.061 ± 0.044, *t* = 1.369, *P* > 0.05; Gammaproteobacteria, β ± SE = −0.536 ± 0.271, *t* = –1.978, *P* = 0.050; linear mixed-effects model) when the effect of diet and individual ID was controlled, we observed a positive effect of age on the abundance of one dominant Firmicutes family (Peptostreptococcaceae β ± SE = 0.478 ± 0.154, *t* = 3.116, *P* = 0.002). We did not observe any obvious influence on the other three abundant families (Enterobacteriaceae, Clostridiaceae_1, and Carnobacteriaceae) and the four main genera (*Escherichia*, *Clostridium_sensu_stricto*, *Plesiomonas*, and *Catellicoccus* (Supplementary Figure 5). As for the effect of diet, addition of fresh loach decreased Enterobacteriaceae abundance, mainly *Escherichia* (β ± SE = −16.028 ± 7.004, *t* = −2.288, *P* = 0.023), however, it increased Carnobacteriaceae abundance, mainly *Catellicoccus* (β ± SE = 12.050 ± 5.136, *t* = 2.346, *P* = 0.020). Diet change had no effect on the two main phyla or the other two abundant families, Peptostreptococcaceae and Clostridiaceae_1.

Sankey plots revealed distinct temporal dynamics when tracking ASVs within the two dominant phyla, Proteobacteria and Firmicutes (Figures 2E,F). A large proportion of the Firmicutes ASVs flew from days 1 to 3 (80%), and then to other time points. Firmicutes ASVs that disappeared reemerged at days 9 and 21 (Figure 2E). New Firmicutes ASVs appeared at most of the time points (10 time points). Compared to Firmicutes ASVs, less Proteobacteria ASVs transferred from day 1 to 3 (55%), and nearly a half of the Proteobacteria ASVs disappeared between days 1 and 3 (Figure 2F). The Proteobacteria ASVs that disappeared reemerged on days 6, 9, 12, and 21. New Proteobacteria ASVs appeared at few time points (seven time points).

**FIGURE 3:**
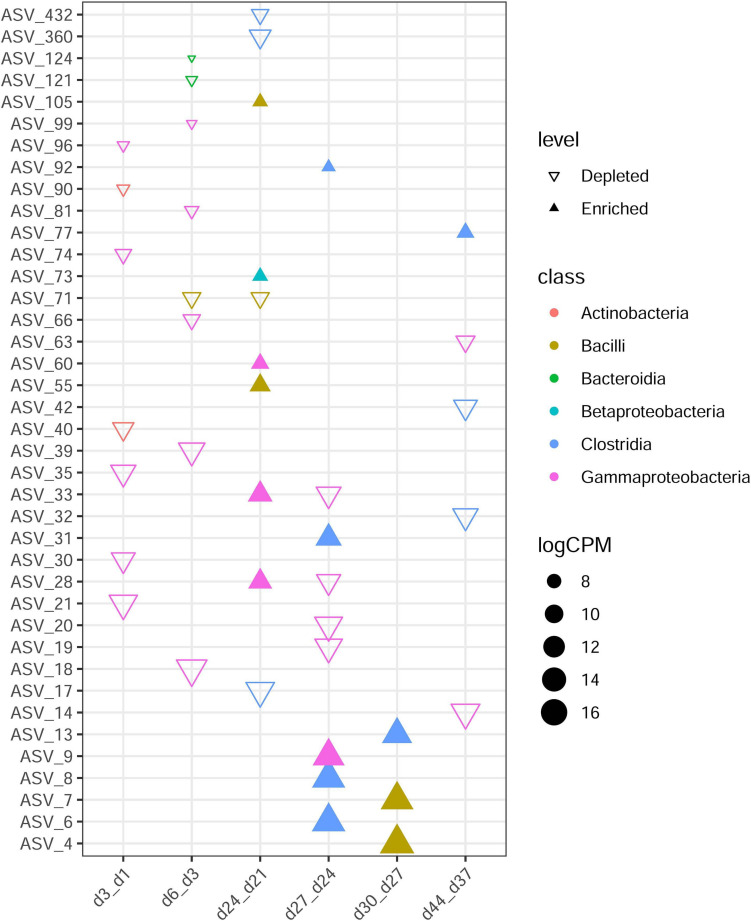
Differences in ASV abundance between two closest ages. The samples of the former time point were taken as control of the latter time point. Each triangle represents a single ASV with adjusted *P* value < 0.05 and | logFC| > 2. Enriched in older age are revealed by filled triangles and hollow triangles shown ASVs enriched in younger age group. ASVs are colored by their class. CPM denotes count per million.

To investigate the colonization and extinction of bacterial groups throughout development in detail, we analyzed the differences between two closest sampling time points (Figure 3). Day 3 showed seven depleted ASVs (total: 137) as compared with day 1, with these from Actinobacteria and Gammaproteobacteria. Comparison of day 6 with day 3 showed eight differential ASVs, with disappearance of Gammaproteobacteria, Bacteroidia, and Bacilli.

Moreover, day 9 versus day 6, day 12 versus day 9, day 15 versus day 12, day 18 versus day 15, day 21 versus day 18, and day 37 versus day 30 comparisons revealed the most similarities in overall ASV abundance (no distinct ASVs were detected), showing no obvious colonization or extinction of bacteria groups from day 6 to day 21 or from day 30 to day 37. The fewest similarities were observed in comparisons between day 24 and day 21 (*n* = 10). Bacilli, Betaproteobacteria, and Gammaproteobacteria were recruited after day 21, and after day 24, more Clostridia than other groups were recruited.

### Gene Functional Pathways During the First Six Weeks

The microbiota of crested ibis was mainly associated with metabolism (mean relative abundance, 44.1%), environmental information processing (18.28%), and functions associated with genetic information processing (16.67%). In addition, the microbiota functional compositions of the most abundant KEGG pathways were stable during development (Supplementary Figure 6).

**FIGURE 4:**
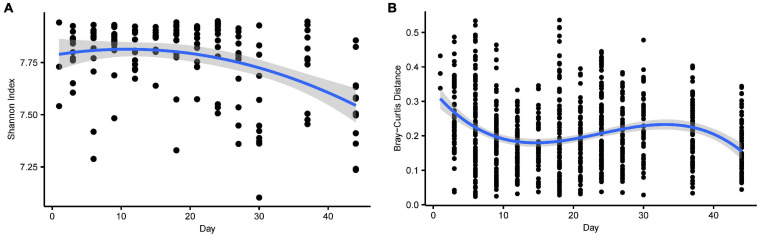
**(A)** The alpha diversity (Shannon index) of gene function over time. The shadow around the lines reveals 95% CI. The alpha diversity was estimated using the Shannon index. **(B)** The beta (Bray-Curtis distance) diversity of gene function over time. The Bray-Curtis distance calculated at the KO level across microbiota from samples collected at the same time point. Higher values denoted more dissimilar gene function.

The alpha diversity of functional profiles decreased during development and fitted a linear and a quadratic age term (Figure 4A and Supplementary Table 2). The dissimilarity in functional profiles within age groups revealed decreasing trends, although they exhibited fluctuation, and fitted a linear and a cubic age term (Figure 4B and Supplementary Table 2).

### Age and the Diet Shaped Gut Microbiota Diversity and Gene Functional Pathways in Crested Ibis

**FIGURE 5:**
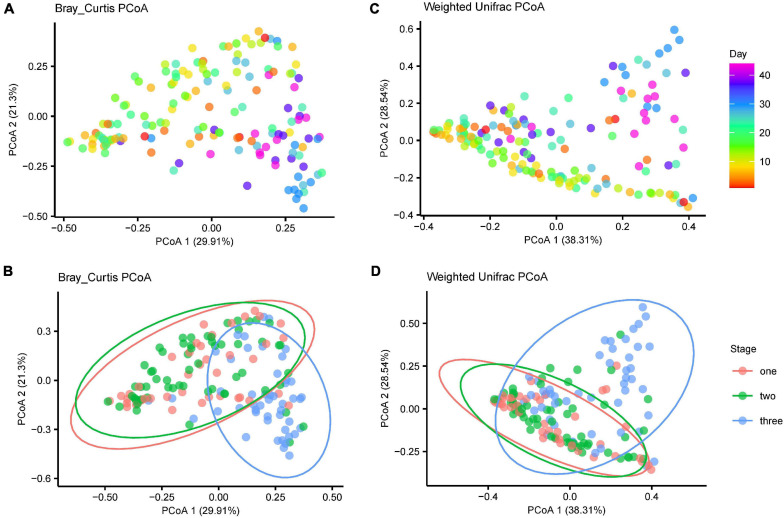
The gut microbiota shift over time. Principle coordinate analysis showing that the gut microbiota with age since birth and stages. **(A)** Bray-Curtis distance for gut microbiota of the age effect **(B)** Bray-Curtis distance for gut microbiota of the three stages **(C)** weighted unifrac distances for gut microbiota of the age effect **(D)** weighted unifrac distances for gut microbiota of the three stages.

The microbiota were highly dynamic during development. With an increase in age, samples within age groups were increased in similarity (Supplementary Figure 1) and tended to cluster by age based on PcoA of both Bray-Curtis and weighted Unifrac distances (Figures 5A,B, PERMANOVA using BC distances: time: *R*^2^ = 0.112, F_12_ = 2.355, *P* = 0.001; Unifrac distance: *R*^2^ = 0.142, F_10_ = 2.696, *P* = 0.001). Samples from stage one to stage two (diet type 1) were separated from samples from stage three (diet type 2) in the first coordinate axis, which suggests diet change was another major factor influencing gut microbiota diversity during development (Figures 5C,D, PERMANOVA using BC distances: *R*^2^ = 0.130, F_2_ = 12.576, *P* = 0.001; Unifrac distance: stage: *R*^2^ = 0.130, F_1_ = 12.343, *P* = 0.001).

Similar temporal structures were observed in the gene functional profiles. Age and diet were also major factors influencing the functional profiles (age: *R*^2^ = 0.101, F_10_ = 1.958, *P* = 0.01; stage: *R*^2^ = 0.188, F_1_ = 18.247, *P* = 0.001; PERMANOVA using BC distances, Supplementary Figure 7).

Sex and genetic relatedness had no significant influence of on either bacteria community structure or functional profiles (*P* > 0.05).

### Specific Genera Participate in Metabolic Pathways

**FIGURE 6:**
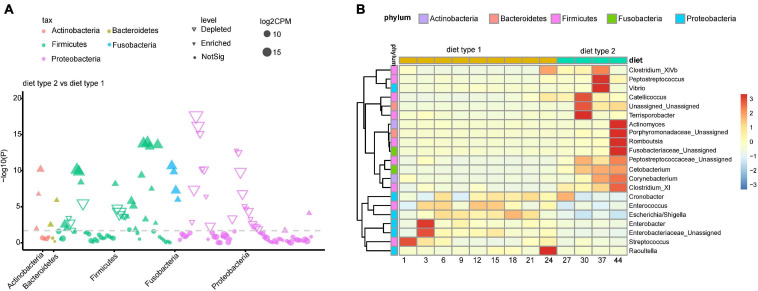
Diet-induced changes in gut microbiota during Crested ibis’ growth. **(A)** Enriched and depleted ASVs for comparison between diet type 2 and diet type 1. **(B)** The relative abundance of enriched and depleted bacteria genus for different diet types along with the age. The shown ASVs and genus was obtained from the differential abundance analysis which was conducted by fitting a generalized linear mixed model with a negative binomial distribution in edgeR. Genus counts were normalized for edgeR size factor.

To reveal diet-related changes in gut microbiota during the growth of crested ibis, we compared raw abundances between diet type 2 and diet type 1 at the ASV and genus levels, respectively, (Figures 6A,B). We detected 29 enriched ASVs and 31 depleted ASVs in diet type 2, with most of the different ASVs coming from Firmicutes and Proteobacteria (Figure 6A). At the genus level, we detected 21 significantly-different bacterial taxa, with 14 enriched in diet type 2 and seven enriched in diet type 1 and belonging to five phyla (Figure 6B). Additionally, the bacterial taxa exhibited obvious temporal variation. The abundances of two Fusobacteria genera, two Bacteroidetes genera, and two Actinobacteria genera were relatively stable across diet type 1 but increased during diet type 2. The other four phyla groups varied across the two diet types.

Among 328 KEGG pathways tested, 10 pathways differed in abundance between diet type 2 and diet type 1 (Supplementary Figure 8), with these including pathways associated with metabolism (8) and organismal systems (2). Four pathways that were all associated with metabolism (one lipid metabolism, one metabolism of terpenoids, and polyketides, and two biosynthesis of other secondary metabolites) were enriched in diet type 2, and the other six pathways were enriched in diet type 1. The abundances of metabolism-associated genes were stable in diet type 2. For diet type1, we observed a decreasing trend for two pathways (Chlorocyclohexane and chlorobenzene degradation, and Fluorobenzoate degradation). At 3 days, three pathways, including one involving immune regulation (the RIG-I-like receptor signaling pathway), one involving the digestive system (Carbohydrate digestion and absorption), and one involving lipid metabolism (Steroid hormone biosynthesis), were abundant.

**FIGURE 7:**
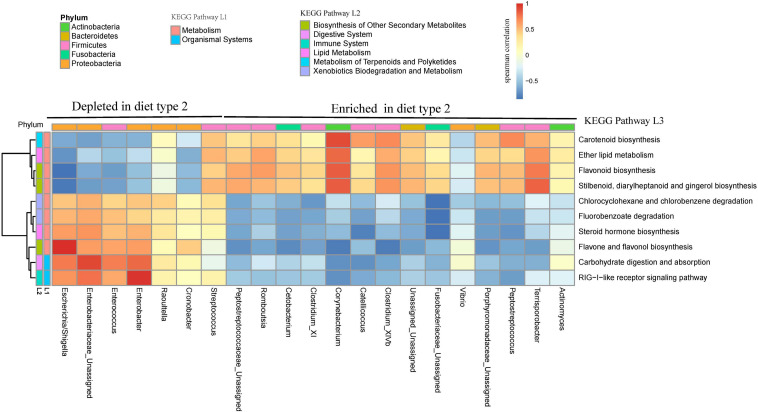
Bacterial taxa from two diet types are related to several KEGG pathways. Gene function of bacteria were predicted using the PICRUST from KEGG annotated databases. Genus and KEGG pathways counts were normalized using edgeR. Spearman’s correlation coefficients were estimated for pairwise comparison between genus abundance and KEGG abundance pathways.

The enriched pathway (lipid metabolism) was highly significant with enriched Firmicutes genera in diet type 2 (Figure 7). The most of depleted pathways in diet type 2 were highly positively significant with depleted Proteobacteria and negatively associated with enriched Firmicutes. For example, carbohydrate digestion and absorption, which was depleted in diet type 2, was positively associated with depleted *Enterobacter* (Proteobacteria) and negatively associated with enriched *Catellicoccus* (Firmicutes).

### Growth Rate Associated With Gut Microbiota Diversity

**FIGURE 8:**
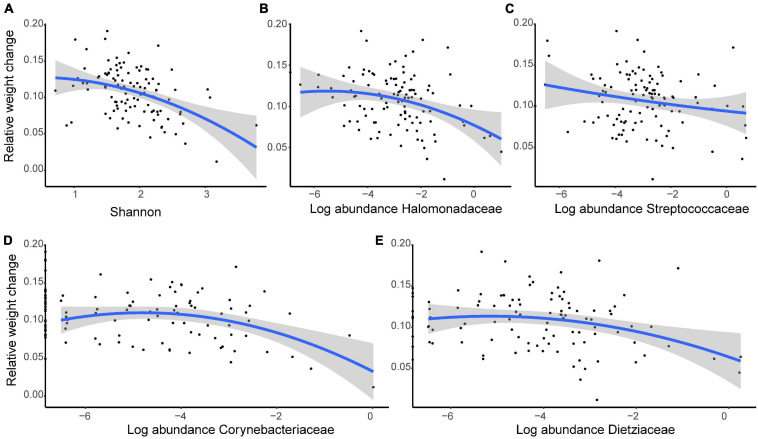
The growth rate of young crested ibis is correlated with gut microbiota. **(A)** Shannon index. The normalized abundance of panel **(B)** Halomonadaceae, **(C)** Streptococcaceae, **(D)** Corynebacteriaceae, and **(E)** Dietziaceae. Relative weight change is measured as the weight change per day (W_t__+__1_–W_t_) against weight at time *t* (W_t_). Age and diet type at time *t* were considered a covariate, and individual ID was controlled as a random factor. The lines represent linear regression lines, while the shaded areas show the 95% confidence interval.

Over the 44-day growth period, we identified a negative relationship between growth rate and alpha diversity when diet and individual ID were controlled (Shannon: β ± SE = −0.020 ± 0.005, *t* = −3.827, *P* < 0.001; Figure 8A). Further analyses of the relative abundances showed that four families (Halomonadaceae, Streptococcaceae, Corynebacteriaceae, and Dietziaceae) were negatively associated with growth during development (Halomonadaceae: β ± SE = −0.019 ± 0.008, *t* = −2.530, *P* = 0.013; Streptococcaceae, β ± SE = −0.020 ± 0.007, *t* = −2.855, *P* = 0.005; Corynebacteriaceae, β ± SE = −0.060 ± 0.024, *t* = −2.496, *P* = 0.014, and Dietziaceae, β ± SE = −0.035 ± 0.014, *t* = −2.397, *P* = 0.019; Figures 8B–E).

## Discussion

Gut microbiota diversity (both alpha and beta) exhibited non-linear changes (Figure 2A) during the sampling period, as expected. Such a pattern was not observed in other age-related studies, including in another study on crested ibis (Ran et al., 2021) and other avian species, due to infrequent sampling. Our findings revealed that bacteria colonization and extinctions occurred throughout the development in crested ibis, and mainly occurred in Proteobacteria and Firmicutes phyla.

The Firmicutes and Proteobacteria phyla dominated the gut microbiota during the first 44 days in crested ibis, with an increase in Firmicutes and a decrease in Proteobacteria. Similar taxonomic changes have been observed in the course of chick development in other birds, such as little penguin (Dewar et al., 2017), arctic shorebirds (Grond et al., 2017), and great tits (Parus major) (Teyssier et al., 2018). Firmicutes produce short-chain fatty acids, which can be absorbed directly by host gut walls as a source of energy (Den Besten et al., 2013) and are positively associated weight gain and immune function in both birds and mammals (Angelakis and Raoult, 2010; Clemente et al., 2012; Liao et al., 2015; John and Mullin, 2016). In particular, an increase in the abundance of Firmicutes was associated with increased Clostridia (mainly Clostridiaceae_1, and Peptostreptococcaceae) and Bacilli (Carnobacteriaceae). Clostridia are also pioneer bacteria in the human gastrointestinal (GI) tract and essential in gut homeostasis (Lopetuso et al., 2013). Bacilli abundance increased considerably at 27 days, which was consistent with the time of fresh loach addition (Supplementary Figure 1). Furthermore, Clostridia and Bacilli were significantly associated with lipid metabolism in the present study (Figure 8, *Catellicoccus*).

Proteobacteria relative abundance in the present study was higher than those in most wild bird species and domestic chicken (Grond et al., 2018). Furthermore, at the class level, Gammaproteobacteria (mainly Enterobacteriaceae) as opposed to alphaproteobacteria were the dominant Proteobacteria taxa in the course of the growth of the young crested ibis, when compared with the dominant bacteria, alphaproteobacterial, in other wild birds (Grond et al., 2018). In particular, Escherichia or Shigella, which are opportunistic pathogens (Strockbine et al., 2015), were the dominant genera within Gammaproteobacteria. However, their high relative abundances in young crested ibis potentially indicate the presence of unknown non-pathogenic functions, such as a key role of diet as a microbial inoculum or involvement in gut immunity and the digestive system.

Notably, in the present study, Fusobacteria (*Cetobacterium* and other unassigned genera; Figure 6) were more dominant from 27 days and enriched in diet type 2 when fresh loach was introduced to chick formula, however, Fusobacteria increased significantly in the Deqing population (another crested ibis’s breeding center in China) from 9 days or earlier, as observed by Ran et al. (2021). Fusobacteria colonization was potentially due to the ingestion of fresh loach, since the time of Fusobacteria colonization is consistent with the addition of fresh loach based on the results of two studies on crested ibis (addition of fresh loach began on day 5 in the Deqing population, personal communication with Ran Jian). The prevalence of Fusobacteria in the gut has been observed in other carnivorous birds (Waite and Taylor, 2015), which suggests its involvement in mutualism between microbiota and hosts, excluding its pathogenic role. One of the Fusobacteria genera, *Cetobacterium*, was also predicted to participate in lipid metabolism in the present study (Figure 8), however, its functions in other avian species require further research. The taxonomic shifts in Firmicutes, Proteobacteria, and Fusobacteria along with the age observed in the present study suggest selective recruitment of specific gut communities by hosts.

Mature gut microbiota showed strong temporal structure and two specific developmental stages. The beginning of the latter stage coincides with the introduction of fresh loach, an increase in the relative abundance of Fusobacteria, and several groups of Firmicutes that both could be involved in lipid metabolism. Furthermore, samples within similar age groups tended to be much more similar than to other age groups with an increase in age, especially from 27 days, which suggests that diet containing fresh loach makes gut community within age groups to converge. Our findings showed that diet and age (along with morphological modifications and immune maturation, Figure 1; Caviedes-Vidal and Karasov, 2001; Killpack et al., 2013) shape gut microbiota during the development of young crested ibis.

When the diet was shifted from diet type 1 to diet type 2 (introduction of fresh loach), more KEGG pathways were identified with more functional including abundant immune, digestive, xenobiotic biodegradation and metabolism, biosynthesis of other secondary metabolites featured in diet type 1 relative to more lip metabolism featured in diet type 2. The higher levels of lipid-metabolism functions in diet type 2 could be attributed to fresh loach requiring more lipid metabolism energy, which may be produced by fresh loach than loach paste (Figure 8). Notably, immune function was enhanced as early as day 3, with more abundant RIG-I-like receptor signaling pathway, which are regulated by Proteobacteria genera such as *Enterobacter*, which suggests that the gut immune system at developed at very early stages of development.

Consistent with previous findings (Gaskins et al., 2002; Dibner and Richards, 2005), in the present study, animal-growth rate was negatively associated with gut bacteria diversity. Four specific taxa (Halomonadaceae, Streptococcaceae, Corynebacteriaceae, and Dietziaceae families from Gammaproteobacteria, Firmicutes, and Actinobacteria phyla) also showed negative effects on the growth rate. A previous studies reported that Streptococcaceae negatively affects ostrich growth (Videvall et al., 2019), however, whether such convergence exists in other wild birds remains to be determined. Additionally, Streptococcaceae and Halomonadaceae are associated with obesity (Garcia-Mantrana et al., 2018) or a high-fat diet (Ijaz et al., 2020), inflammation (Zeng et al., 2016), and other diseases (Chen et al., 2011) in rodents and humans, however, few studies exist on the function of two Actinobacteria families (Corynebacteriaceae and Dietziaceae). These findings suggest conserved interactions between hosts and vertebrate gut microbiota.

Sampling at crested ibis hatching already revealed diverse gut microbiota (Figure 2A), which are consistent with previous observations (Ran et al., 2021). According to the results, crested ibis could acquire microbiota when inside the egg and bacterial colonization occurred before hatching. Considering the eggs in the present study were hatched artificially without mother crested ibis indicate that the microbes may enter the GI of crested ibis embryos via penetration through eggshell pores and embryonic membranes after laying (Gantois et al., 2009; Martelli and Davies, 2012). The colonization of gut microbiota before hatching might result in immunological and metabolic advantages for young crested ibis (Grond et al., 2017). Initial gut microbiota colonization took place earlier than the crested ibis could hatch, hence research on crested ibis embryos is warranted.

In conclusion, the present study showed that gut microbiota diversity displayed non-linear changes during the early stages of development of crested ibis, with multiple shifts occurring mainly in Proteobacteria and Firmicutes. The study also provides evidence that both diet and age influence microbiota structure. Microbiota changes observed were correlated with host growth and could influence host fitness over the long term. Our findings and those of studies on humans (de Muinck and Trosvik, 2018) both highlight the importance of frequent sampling strategies, when studying microbiome development during the early stages of development of vertebrates. Gut microbiota diversity could increase after day 44 when young birds move outdoors and are exposed to new diverse environments. Specially, Fusobacteria abundance significantly increases as crested ibis are fed with fresh loach. Further studies on the establishment of gut microbiota after 6 weeks to 1 year would better explain gut microbiota convergence in crested ibis.

## Data Availability Statement

The raw sequence data reported in this paper have been deposited in the Genome Sequence Archive (Genomics, Proteomics, and Bioinformatics 2017) in National Genomics Data Center (Nucleic Acids Research 2021), China National Center for Bioinformation/Beijing Institute of Genomics, Chinese Academy of Sciences, under accession number CRA004274 that are publicly accessible at https://bigd.big.ac.cn/gsa.

## Ethics Statement

The animal study was reviewed and approved by Decision on Animal Ethics from Sichuan Provincial Academy of Natural Resource Sciences.

## Author Contributions

HQY and YDL collected the sample. YZ conducted the experiment, data analysis, and drafted the manuscript. YZ and KYT conceived of the idea and designed the study. KYT and KH participated in drafting the manuscript. All authors contributed to the article and approved the submitted version.

## Conflict of Interest

The authors declare that the research was conducted in the absence of any commercial or financial relationships that could be construed as a potential conflict of interest.

## Publisher’s Note

All claims expressed in this article are solely those of the authors and do not necessarily represent those of their affiliated organizations, or those of the publisher, the editors and the reviewers. Any product that may be evaluated in this article, or claim that may be made by its manufacturer, is not guaranteed or endorsed by the publisher.

## Footnotes

1
www.drive5.com/sintax/

2
www.ehbio.com
