# Supplementary figures and images for: Establishment of Gut Microbiome During Early Life and Its Relationship With Growth in Endangered Crested Ibis (Nipponia nippon)
Source: Front Microbiol. 2021 Aug 9;12:723682. doi: 10.3389/fmicb.2021.723682 (PMC8382091; doi:10.3389/fmicb.2021.723682)

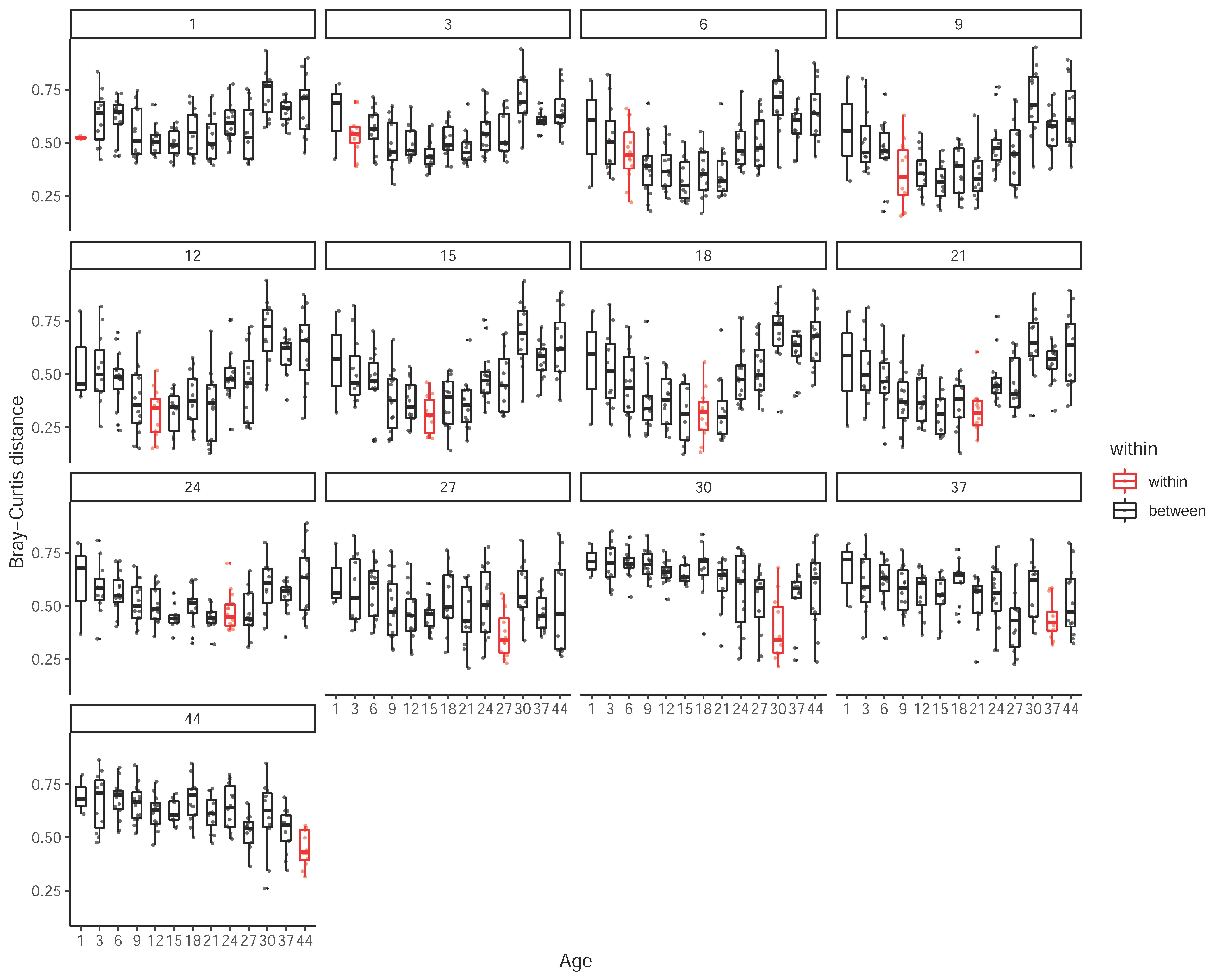

Supplement: Supplementary file 1 [file Image_1.JPEG]

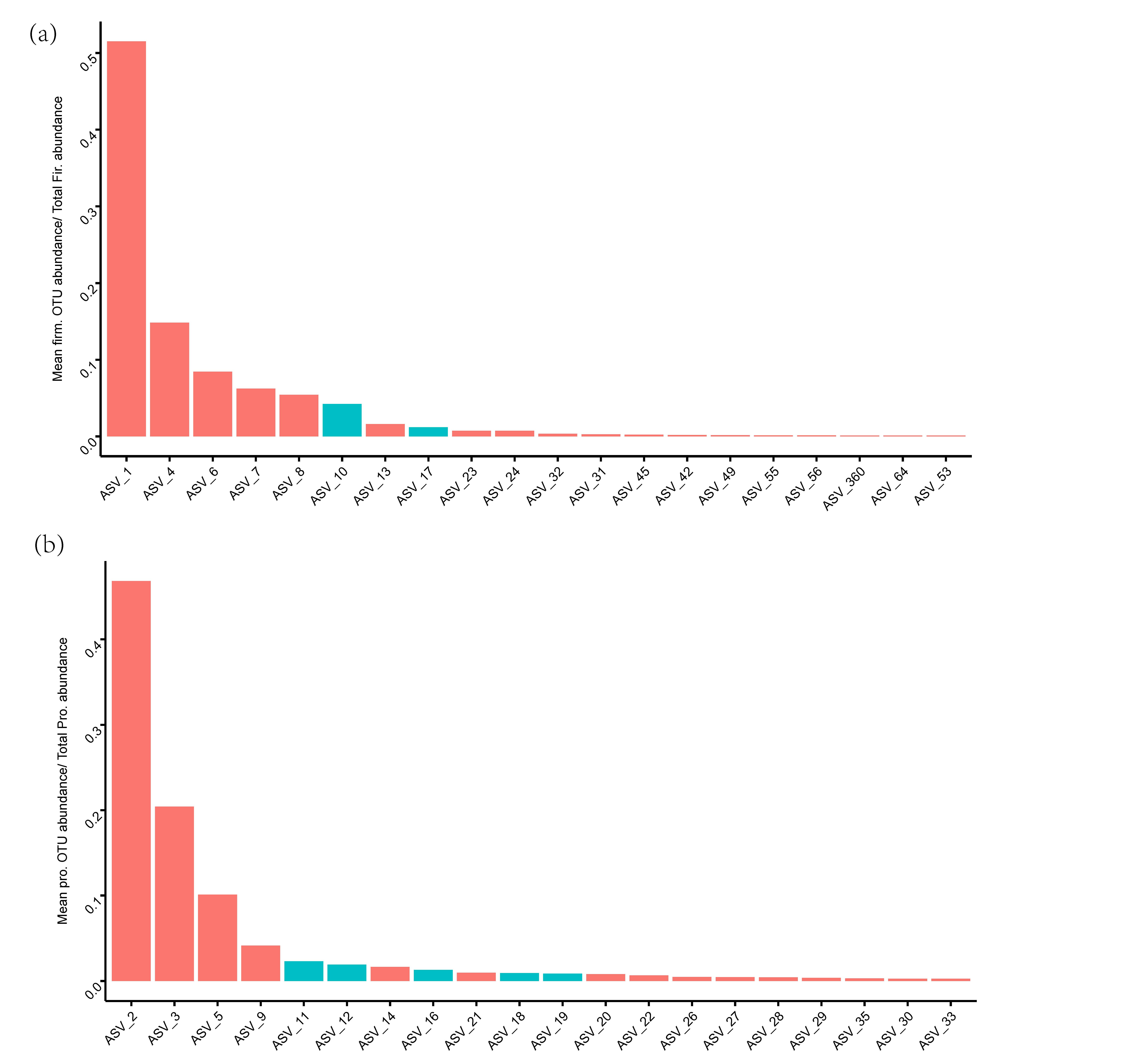

Supplement: Supplementary file 2 [file Image_2.JPEG]

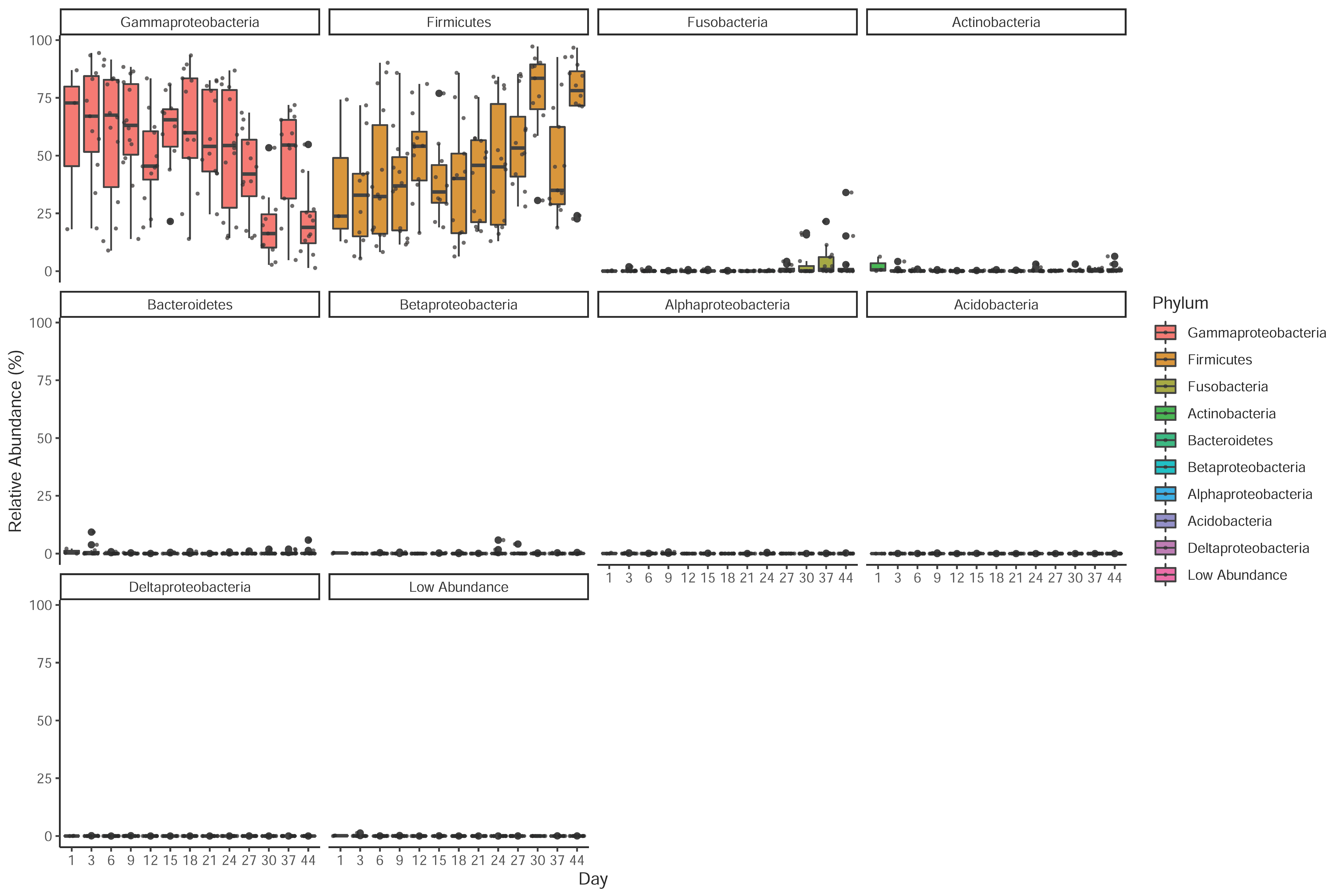

Supplement: Supplementary file 3 [file Image_3.JPEG]

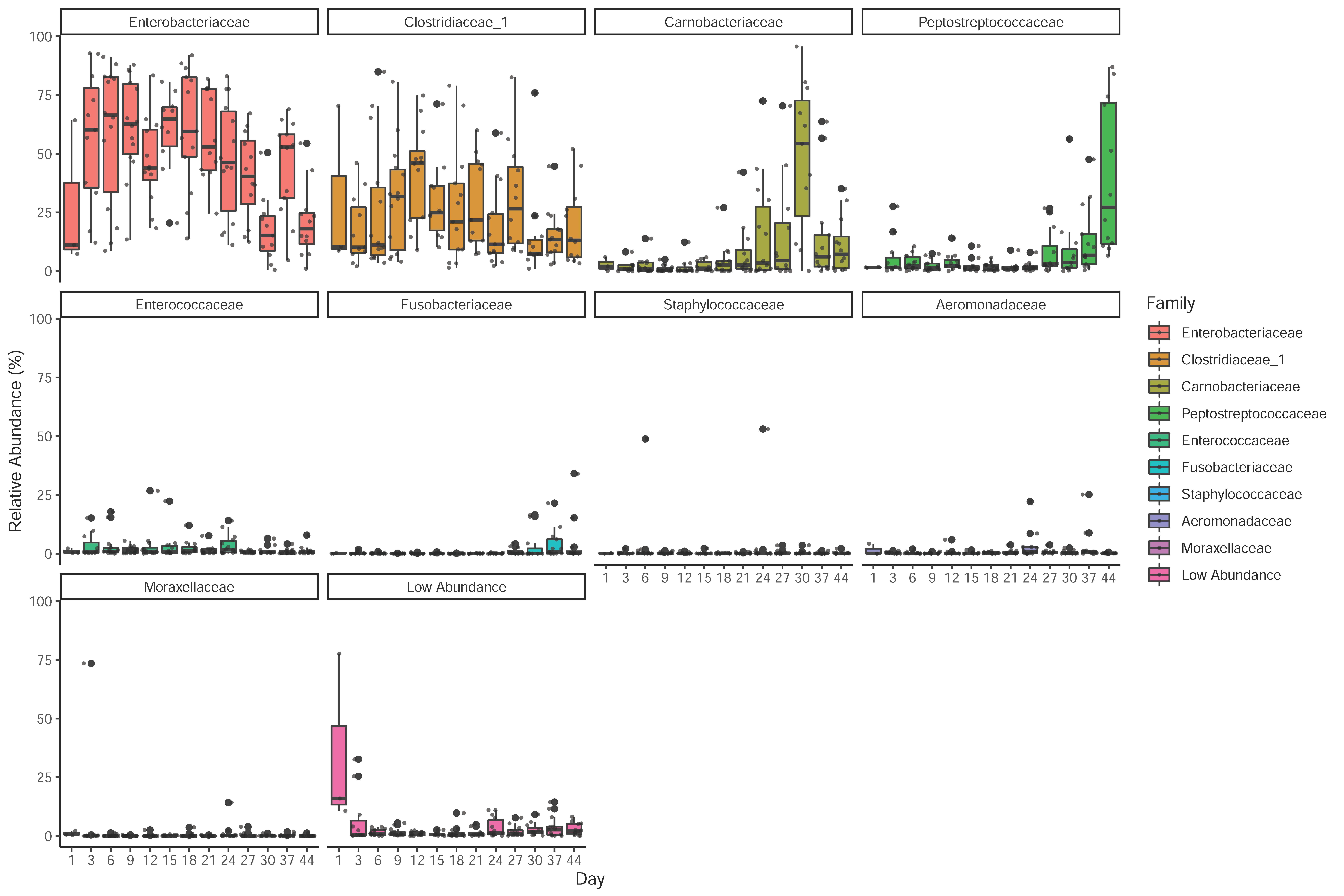

Supplement: Supplementary file 4 [file Image_4.JPEG]

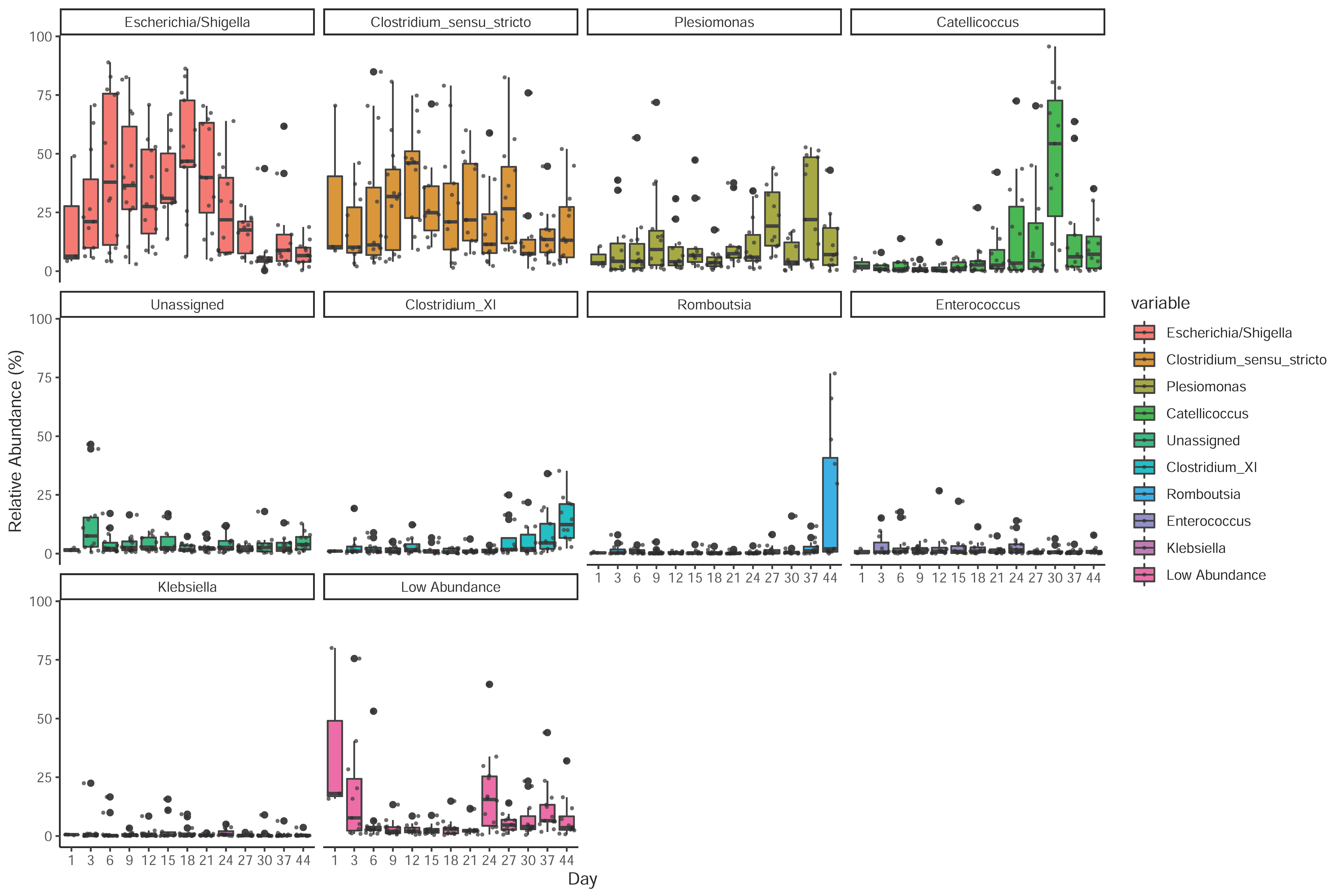

Supplement: Supplementary file 5 [file Image_5.JPEG]

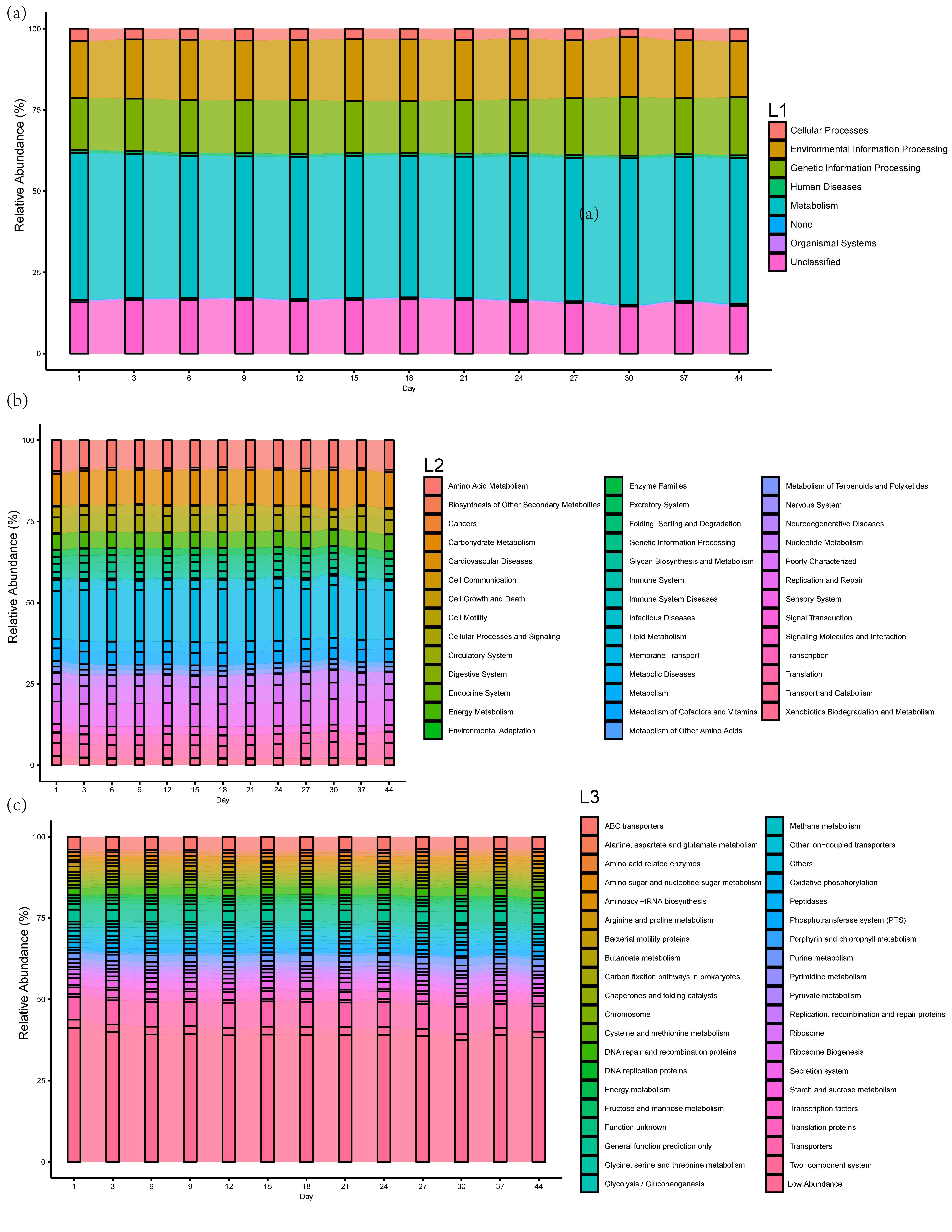

Supplement: Supplementary file 6 [file Image_6.JPEG]

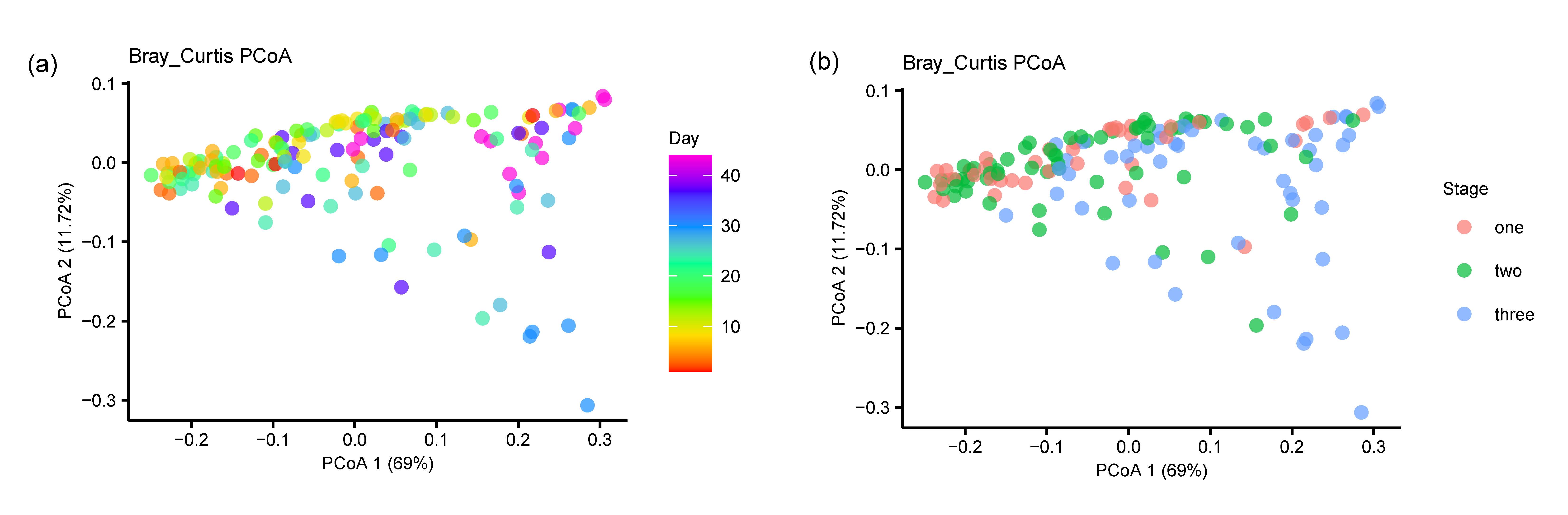

Supplement: Supplementary file 7 [file Image_7.JPEG]

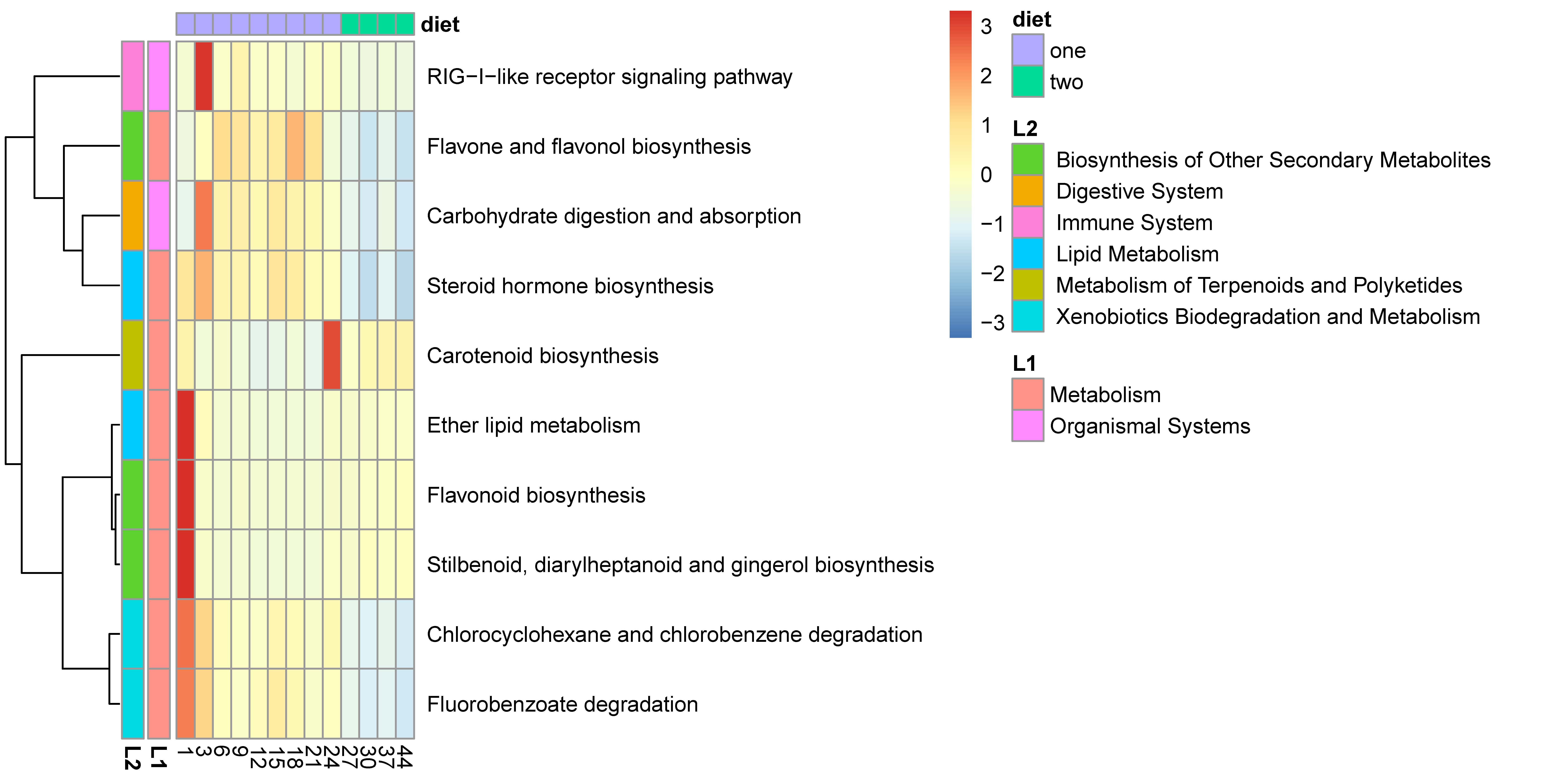

Supplement: Supplementary file 8 [file Image_8.JPEG]
