# Supplementary material for: Establishment of Gut Microbiome During Early Life and Its Relationship With Growth in Endangered Crested Ibis (Nipponia nippon)
Source: Front Microbiol. 2021 Aug 9;12:723682. doi: 10.3389/fmicb.2021.723682 (PMC8382091; doi:10.3389/fmicb.2021.723682)
Supplement: Supplementary file 10 [file Data_Sheet_3.docx]

Establishment of gut microbiome during early life and its relationship with growth in endangered crested ibis (*Nipponia nippon*)

^1^ Institute of Qinghai-Tibetan Plateau, Southwest Minzu University, Chengdu, Sichuan Province, China

^2^ Sichuan Province Laboratory for Natural Resources Protection and Sustainable Utilization, Sichuan Provincial Academy of Natural Resource Sciences, Chengdu, China

^3^ Emei Breeding Center for Crested Ibis, Emei, Sichuan Province, China

^4^ College of Animal Sciences & Technology, Zhejiang A & F University, Hangzhou, China

^5^ College of Life Science, Sichuan Normal University, Chengdu, Sichuan Province, China

***** Corresponding author**:** Ying Zhu and Keyi Tang

**Emails:**

Ying Zhu, so_zy2003@126.com

Yudong Li, animal_resource@163.com

Haiqiong Yang, sw_laoyang@163.com

Ke He**,** heke@zafu.edu.cn

Keyi Tang, tangkeyi0214@163.com

Supplementary Material

Supplementary data S1. The script for fitting linear mixed model of growth rare to alpha diversity and specific microbial taxa.

Supplementary data S2. The script for differential analysis using a negative binomial generalized linear model.

**Supplementary Figure 1.** Beta diversity as estimated using the Bray-Curtis distance of gut microbiota within and between groups. The header shows the age in days while the x-axes display all age comparisons. Within age groups are indicated by red.

**Supplementary Figure 2.** ASV proportions within Firmicutes (a) and Proteobacteria (b). The relative abundance ratio of the top 20 ASVs from Firmicutes and Proteobacteria to the total relative abundance of Firmicutes and Proteobacteria, respectively. Blue bars indicate ASVs with a significant correlation with mean contemporaneous Bray-Curtis distances (Table S3). Fir., Firmicutes; Pro., Proteobacteria. The phylum of Firmicutes was dominated by *Clostridium_sensu_stricto* (ASV_1, 51.5% of total Firmicutes abundance), while the phylum of Proteobacteria was dominated by Escherichia/Shigella (ASV_2, 46.8% of total Proteobacteria).

**Supplementary Figure 3.** The relative abundance of the top 10 bacteria phyla (class) for gut microbiota in crested ibis change over time. Different groups display different trends with the increasing age.

**Supplementary Figure 4.** The relative abundance of the top 10 bacteria family for gut microbiota in crested ibis change over time. Different tax groups display different trends with the increasing age.

**Supplementary Figure 5.** The relative abundance of the top 10 bacteria genera for gut microbiota in crested ibis change over time. Different tax groups display different trends with the increasing age.

**Supplementary Figure 6.** The relative abundance of the gene functional pathways for gut bacteria during growth in crested ibis. Bacterial gene function was predicted using the PICRUST from KEGG annotated databases. (a) L1 levels (b) L2 levels (c) top 40 of the L3 levels.

**Supplementary Figure 7.** The gene function structure of gut microbiota based on principle coordinate analysis. (a) Bray-Curtis distance for gene function of age effect (b) Bray-Curtis distance for gene function of three stages.

**Supplementary Figure 8.** The relative abundance of enriched and depleted KEGG pathways for comparison between diet type 2 and diet type 1 along with the age. The differential KEGG pathways were obtained from differential abundance analysis which was conducted by fitting a generalized linear model with a negative binomial distribution in edgeR. KEGG pathways counts were normalized for edgeR size factor.

**Table S1.** The detailed information of samples used in this study.

**Table S2.** Polynomial linear model of alpha and beta diversity against age.

|  |  |  | Estimate | Std. Error | t value | *P* |
| --- | --- | --- | --- | --- | --- | --- |
| ASV | Shannon | (Intercept) | 2.216 | 0.166 | 13.313 | 0.000 |
|  |  | day | -0.102 | 0.035 | -2.935 | 0.004 |
|  |  | I(day^2) | 0.006 | 0.002 | 3.042 | 0.003 |
|  |  | I(day^3) | 0.000 | 0.000 | -2.971 | 0.004 |
|  |  | Stage two | -0.155 | 0.167 | -0.927 | 0.356 |
|  | Bray-Curtis | (Intercept) | 0.579 | 0.014 | 40.798 | < 0.001 |
|  |  | day | 1.863 | 0.375 | 4.966 | < 0.001 |
|  |  | I(day^2) | 1.755 | 0.196 | 8.972 | < 0.001 |
|  |  | I(day^3) | -1.940 | 0.219 | -8.855 | < 0.001 |
|  |  | Stage two | -0.151 | 0.030 | -5.064 | < 0.001 |
| KO | Shannon | (Intercept) | 7.787 | 0.023 | 335.344 | < 0.001 |
|  |  | day | -0.415 | 0.295 | -1.406 | 0.162 |
|  |  | I(day^2) | -0.393 | 0.170 | -2.308 | 0.023 |
|  |  | Stage two | -0.088 | 0.051 | -1.705 | 0.090 |
|  | Bray-Curtis | (Intercept) | 0.218 | 0.006 | 34.622 | < 0.001 |
|  |  | day | 0.162 | 0.212 | 0.767 | 0.444 |
|  |  | I(day^2) | 0.189 | 0.110 | 1.714 | 0.087 |
|  |  | I(day^3) | -0.905 | 0.123 | -7.330 | < 0.001 |
|  |  | Stage two | -0.039 | 0.017 | -2.331 | 0.020 |

**Table S3.** Correlation between mean relative abundance of top 20 ASVs and mean contemporaneous Bray-Curtis distances between individuals. The relationship was estimated between ASVs indicated and the mean contemporaneous Bray-Curtis distances between individuals.

|  | Spearman | *P* | Phylum | Class | Order | Family | Genus |
| --- | --- | --- | --- | --- | --- | --- | --- |
| ASV_1 | -0.390 | 0.188 | Firmicutes | Clostridia | Clostridiales | Clostridiaceae_1 | Clostridium_sensu_stricto |
| ASV_2 | -0.550 | 0.052 | Proteobacteria | Gammaproteobacteria | Enterobacteriales | Enterobacteriaceae | Escherichia/Shigella |
| ASV_3 | 0.370 | 0.216 | Proteobacteria | Gammaproteobacteria | Enterobacteriales | Enterobacteriaceae | Plesiomonas |
| ASV_4 | 0.780 | **0.002** | Firmicutes | Bacilli | Lactobacillales | Carnobacteriaceae | Catellicoccus |
| ASV_5 | -0.620 | **0.025** | Proteobacteria | Gammaproteobacteria | Enterobacteriales | Enterobacteriaceae | Escherichia/Shigella |
| ASV_6 | 0.580 | **0.039** | Firmicutes | Clostridia | Clostridiales | Peptostreptococcaceae | Clostridium_XI |
| ASV_7 | 0.780 | 0.002 | Firmicutes | Bacilli | Lactobacillales | Carnobacteriaceae | Catellicoccus |
| ASV_8 | 0.530 | 0.061 | Firmicutes | Clostridia | Clostridiales | Peptostreptococcaceae | Romboutsia |
| ASV_9 | 0.360 | 0.231 | Proteobacteria | Gammaproteobacteria | Enterobacteriales | Enterobacteriaceae | Plesiomonas |
| ASV_10 | -0.490 | 0.090 | Firmicutes | Bacilli | Lactobacillales | Enterococcaceae | Enterococcus |
| ASV_11 | -0.720 | **0.006** | Proteobacteria | Gammaproteobacteria | Enterobacteriales | Enterobacteriaceae | Unassigned |
| ASV_12 | -0.490 | 0.086 | Proteobacteria | Gammaproteobacteria | Enterobacteriales | Enterobacteriaceae | Enterobacter |
| ASV_14 | 0.480 | 0.099 | Proteobacteria | Gammaproteobacteria | Aeromonadales | Aeromonadaceae | Aeromonas |
| ASV_13 | 0.130 | 0.668 | Firmicutes | Clostridia | Clostridiales | Peptostreptococcaceae | Unassigned |
| ASV_15 | 0.710 | **0.006** | Fusobacteria | Fusobacteriia | Fusobacteriales | Fusobacteriaceae | Cetobacterium |
| ASV_16 | -0.270 | 0.374 | Proteobacteria | Gammaproteobacteria | Enterobacteriales | Enterobacteriaceae | Unassigned |
| ASV_17 | -0.410 | 0.162 | Firmicutes | Clostridia | Clostridiales | Clostridiaceae_1 | Clostridium_sensu_stricto |
| ASV_21 | 0.120 | 0.707 | Proteobacteria | Gammaproteobacteria | Pseudomonadales | Pseudomonadaceae | Pseudomonas |
| ASV_18 | -0.190 | 0.541 | Proteobacteria | Gammaproteobacteria | Pseudomonadales | Moraxellaceae | Acinetobacter |
| ASV_19 | -0.420 | 0.150 | Proteobacteria | Gammaproteobacteria | Enterobacteriales | Enterobacteriaceae | Klebsiella |
