# Supplementary material for: Establishment of Gut Microbiome During Early Life and Its Relationship With Growth in Endangered Crested Ibis (Nipponia nippon)
Source: Front Microbiol. 2021 Aug 9;12:723682. doi: 10.3389/fmicb.2021.723682 (PMC8382091; doi:10.3389/fmicb.2021.723682)
Supplement: Supplementary file 11 [file Data_Sheet_1.PDF]

```
# This script is for investigating the effect of alpha diversity and
# specific microbial taxa on growth rate of young crested ibis.


rm(list=ls()) # clean environment object


# Design of experiment
design = read.table("metadata_weight.txt", header=T, row.names= 1,
sep="\t")

# add a column for showing environment changes
design$phase <- design$Day
design$phase[design$Day<12] <- "p1"  #stage one in Figure 1
design$phase[design$Day%in%c(12,15,18,21,24)] <- "p2"  #stage two in Figure
1
design$phase[design$Day>24] <- "p3" #stage three in Figure 1

# alpha diversity
vegan = read.table("vegan.txt", header=T, row.names= 1, sep="\t")
vegan <- vegan[rownames(design),]
rownames(design)==rownames(vegan)

# combine data
dat <- data.frame(vegan, group=design$Group,
day=design$Day,day2=design$Day2,
                  sex=design$Sex, stage=design$Stage,
individual=design$Individual,
                  weight=design$weight, phase=design$phase)


# growth rate against alpha diversity
#----------------data preparation---------------------------
# calculate the growth rate
dat.t <- t(dat)
sub_design <- design[,c("Day2", "Individual", "weight") ]
str(sub_design)
sub_design$Day2 <- factor(sub_design$Day2, level=c("d1","d3", "d6", "d9",
"d12",
                                                   "d15", "d18","d21",
"d24" , "d27",                                                  "d30",
"d37", "d44"))
head(sub_design)

library(reshape2)

indi_growth <- dcast(sub_design, Individual~Day2)
str(indi_growth)

indi_growth$D1 <- (indi_growth$d3-indi_growth$d1)/(2*indi_growth$d1)
indi_growth$D3 <- (indi_growth$d6-indi_growth$d3)/(3*indi_growth$d3)
indi_growth$D6 <- (indi_growth$d9-indi_growth$d6)/(3*indi_growth$d6)
indi_growth$D9 <- (indi_growth$d12-indi_growth$d9)/(3*indi_growth$d9)
indi_growth$D12 <- (indi_growth$d15-indi_growth$d12)/(3*indi_growth$d12)
indi_growth$D15 <- (indi_growth$d18-indi_growth$d15)/(3*indi_growth$d15)
indi_growth$D18 <- (indi_growth$d21-indi_growth$d18)/(3*indi_growth$d18)
indi_growth$D21 <- (indi_growth$d24-indi_growth$d21)/(3*indi_growth$d21)
indi_growth$D24 <- (indi_growth$d27-indi_growth$d24)/(3*indi_growth$d24)
```

```
indi_growth$D27 <- (indi_growth$d30-indi_growth$d27)/(3*indi_growth$d27)
indi_growth$D30 <- (indi_growth$d37-indi_growth$d30)/(7*indi_growth$d30)
indi_growth$D37 <- (indi_growth$d44-indi_growth$d37)/(7*indi_growth$d37)

growth_rate <- melt(indi_growth[,-(2:14)],)
growth_rate$sample <- paste0(growth_rate$Individual,growth_rate$variable,
sep="")
colnames(growth_rate)[3] <- "g_rate"

rownames(growth_rate) <- growth_rate$sample

all.growth <- merge(vegan, growth_rate, by="row.names", all=T)
rownames(all.growth) <- all.growth$Row.names
all.growth <- all.growth[,-1]
all.growth <- all.growth[,!colnames(all.growth)=="Individual"]
all.growth <- all.growth[,!colnames(all.growth)=="variable"]
all.growth <- all.growth[,!colnames(all.growth)=="sample"]


all.growth <- all.growth[rownames(all.growth)%in%rownames(design),]

#all.growth+design
all.growth <- merge(all.growth, design, by="row.names", all=T)
rownames(all.growth) <- all.growth$Row.names
all.growth <- all.growth[,-1]


#---------------merge top 15 families ----------

#  raw reads count of each OTU in each sample. Mean abundance >=0.01%
otu_table <- read.table("count_8OTU.txt", row.names= 1,  header=T,
sep="\t")#get the count matrix

#  taxonomy for each OTU
taxonomy = read.delim("taxonomy.txt", row.names= 1,header=F, sep="\t")
colnames(taxonomy) =
c("kingdom","phylum","class","order","family","genus","species")

taxonomy <- taxonomy[-1,]
#cross  filtering
taxonomy <- taxonomy[rownames(otu_table),]
tax_count = merge(taxonomy, otu_table, by="row.names")


tax_count_sum = aggregate(tax_count[,-(1:8)], by=tax_count[6], FUN=sum) #
mean

rownames(tax_count_sum) = tax_count_sum$family

tax_count_sum = tax_count_sum[,-1]

per = t(t(tax_count_sum)/colSums(tax_count_sum,na=T)) * 100 # normalization
to total 100

mean_sort = per[(order(-rowSums(per))), ] # decrease sort
colSums(mean_sort)

mean_sort=as.data.frame(mean_sort)
```

```
other = colSums(mean_sort[16:dim(mean_sort)[1], ])
mean_sort = mean_sort[1:(16-1), ]
mean_sort = rbind(mean_sort,other)
rownames(mean_sort)[16] = c("Low Abundance")
mean_sort <- mean_sort[,rownames(design)]
mat <- t(mean_sort)

all.growth <- merge(all.growth, mat, by="row.names")
rownames(all.growth) <- all.growth$Row.names
all.growth <- all.growth[,-1]

#------------------fit the model

#linear model for checking the effect of single variables
# family group effect
all.growth.sub <- all.growth
#family group
lm <- lm(g_rate~Group, data=all.growth.sub)
summary(lm)

#sex
lm_sex <- lm(g_rate~Sex, data=all.growth.sub)
summary(lm_sex)


#age
lm_day <- lm(g_rate~Day, data=all.growth.sub)
summary(lm_day)#significant

ggplot(all.growth.sub, aes(x=Day, y=g_rate))+
  geom_point()

# chick weight at time (t-1)
lm_weight <- lm(g_rate~weight, data=all.growth.sub)
summary(lm_weight)#significant

# diet types
lm_stage <- lm(g_rate~Stage, data=all.growth.sub)
summary(lm_stage)#significant

ggplot(all.growth.sub, aes(x=Stage, y=g_rate))+
  geom_boxplot()

# temperature effect
lm_stage <- lm(g_rate~phase, data=all.growth.sub)
summary(lm_stage)#significant

ggplot(all.growth.sub, aes(x=phase, y=g_rate))+
  geom_boxplot()


#weight of food
lm_food <- lm(g_rate~food, data=all.growth.sub)
summary(lm_food)#negative


#Individual ID
lm_ind <- lm(g_rate~Individual, data=all.growth.sub)
```

```
summary(lm_ind)

# age, chick weight, diet, environment changes, food weight and individual
ID all have effects on growth rate


#----------relation between independent variables---------
#age~weight
if(!require(Hmisc))install.packages("Hmisc")
library("Hmisc")
rcorr(all.growth.sub$Day, all.growth.sub$weight, type="spearman")#正相关

ggplot(all.growth.sub, aes(x=Day, y=weight))+
  geom_point()
# positive related

#age~food
rcorr(all.growth.sub$Day, all.growth.sub$food, type="spearman")#正相关
ggplot(all.growth.sub, aes(x=Day, y=food))+
  geom_point()
# positive related


#food~weight
rcorr(all.growth.sub$weight, all.growth.sub$food, type="spearman")#正相关
ggplot(all.growth.sub, aes(x=weight, y=food))+
  geom_point()
# positive related


# there is strong positive linear relationship among age, chick weight at
time t-1, and food weight


if(!require(car)) install.packages("car")

library(car)# check the multicollinearity using variance inflation factors

lm <- lm(g_rate~Individual+Day+weight+food+Stage, data=all.growth.sub)
summary(lm)
vif(lm) # remove food

lm<- lm(g_rate~Individual+Day+weight+Stage, data=all.growth.sub)
summary(lm)#remove chick weight variable
vif(lm)

lm<- lm(g_rate~Individual+Day+Stage, data=all.growth.sub)
summary(lm)
vif(lm)
# keep individual+day+stage

# install and load packages
if(!require(nlme))install.packages("nlme")
library(nlme)

all.growth.sub <- na.omit(all.growth)

#------------shannon index vs growth rate-------------
```

```
lme <- lme(g_rate~shannon+Day+Stage,
            random=~1|Individual,method ="ML",data=all.growth.sub)
summary(lme)



#------------------Family taxa vs growth rate-----------

# Enterobacteriaceae, top.1
lme_f_enter <- lme(g_rate~Enterobacteriaceae+Day+Stage,
                   random=~1|Individual,method ="ML",data=all.growth.sub)
summary(lme_f_enter)

# Clostridiaceae_1, top.2

lme_f_clo <- lme(g_rate~Clostridiaceae_1+Day+Stage,
                 random=~1|Individual,method ="ML",data=all.growth.sub)
summary(lme_f_clo)

#Carnobacteriaceae, top 3

lme_f_car <- lme(g_rate~Carnobacteriaceae+Day+Stage,
                 random=~1|Individual,method ="ML",data=all.growth.sub)
summary(lme_f_car)


#Peptostreptococcaceae, top 4

lme_f_pep <- lme(g_rate~Peptostreptococcaceae+Day+Stage,
                 random=~1|Individual,method ="ML",data=all.growth.sub)
summary(lme_f_pep)


#Enterococcaceae,  top 5
lme_f_enter <- lme(g_rate~Enterococcaceae+Day+Stage,
                   random=~1|Individual,method ="ML",data=all.growth.sub)
summary(lme_f_enter)


#Fusobacteriaceae, top 6

lme_f_fuso <- lme(g_rate~Fusobacteriaceae+Day+Stage,
                  random=~1|Individual,method ="ML",data=all.growth.sub)
summary(lme_f_fuso)



#Staphylococcaceae, top 7
lme_f_sta <- lme(g_rate~Staphylococcaceae+Day+Stage,
                 random=~1|Individual,method ="ML",data=all.growth.sub)
summary(lme_f_sta)


#Aeromonadaceae, top 8
lme_f_aer <- lme(g_rate~Aeromonadaceae+Day+Stage,
                 random=~1|Individual,method ="ML",data=all.growth.sub)
summary(lme_f_aer)
```

```
#Moraxellaceae, top 9

lme_f_mor <- lme(g_rate~Moraxellaceae+Day+Stage,
                 random=~1|Individual,method ="ML",data=all.growth.sub)
summary(lme_f_mor)

#Pseudomonadaceae, top 10

lme_f_pse <- lme(g_rate~Pseudomonadaceae+Day+Stage,
                 random=~1|Individual,method ="ML",data=all.growth.sub)
summary(lme_f_pse)


#Lachnospiraceae, top 11

lme_f_lac <- lme(g_rate~Lachnospiraceae+Day+Stage,
                 random=~1|Individual,method ="ML",data=all.growth.sub)
summary(lme_f_lac)


#Halomonadaceae, top 12
lme_f_hal <- lme(g_rate~Halomonadaceae+Day+Stage,
                 random=~1|Individual,method ="ML",data=all.growth.sub)
summary(lme_f_hal)   #negative   Gammaproteobacteria


#Streptococcaceae, top 13
lme_f_str <- lme(g_rate~Streptococcaceae+Day+Stage,
                 random=~1|Individual,method ="ML",data=all.growth.sub)
summary(lme_f_str)  #negative   Firmicutes


#Corynebacteriaceae, top 14
lme_f_cor <- lme(g_rate~Corynebacteriaceae+Day+Stage,
                 random=~1|Individual,method ="ML",data=all.growth.sub)
summary(lme_f_cor) # negative, Actinobacteria


#Dietziaceae, top 15
lme_f_die <- lme(g_rate~Dietziaceae+Day+Stage,
                 random=~1|Individual,method ="ML",data=all.growth.sub)
summary(lme_f_die) #negative, Actinobacteria


#---------------------plot------------------------------
# install and load packages
if(!require(ggplot2))install.packages("ggplot2")
library(ggplot2)

# alpha diversity
p=ggplot(all.growth.sub,aes(x=simpson,y=g_rate))+
  labs(x="Simpson", y="Relative weight change") + theme_classic()+
  geom_point(size=1,alpha=1)
p1=p+geom_smooth()
p2=p+geom_smooth(method = "lm", formula = y ~ poly(x,2))
```

```
p1
p2
p_simpson_growth <- p2

p=ggplot(all.growth.sub,aes(x=shannon,y=g_rate))+
  labs(x="Shannon", y="Relative weight change") + theme_classic()+
  geom_point(size=1,alpha=1)
p1=p+geom_smooth()
p2=p+geom_smooth(method = "lm", formula = y ~ poly(x,2))
p1
p2
p_shannon_growth <- p2


#----------------specific taxa-----------------------

p=ggplot(all.growth.sub,aes(x=log(Halomonadaceae),y=g_rate))+
  labs(x="Log abundance Halomonadaceae ", y="Relative weight change") +
theme_classic()+
  geom_point(size=1,alpha=1)
p1=p+geom_smooth()
p2=p+geom_smooth(method = "lm", formula = y ~ poly(x,2))
p3=p+geom_smooth(method="lm")
p1
p2
p3
p_Halomonadaceae_growth <- p2

p=ggplot(all.growth.sub,aes(x=log(Streptococcaceae),y=g_rate))+
  labs(x="Log abundance Streptococcaceae ", y="Relative weight change") +
theme_classic()+
  geom_point(size=1,alpha=1)
p1=p+geom_smooth()
p2=p+geom_smooth(method = "lm", formula = y ~ poly(x,2))
p3=p+geom_smooth(method="lm")
p
p2
p3
p_Streptococcaceae_growth <- p2


p=ggplot(all.growth.sub,aes(x=log(Corynebacteriaceae),y=g_rate))+
  labs(x="Log abundance Corynebacteriaceae ", y="Relative weight change") +
theme_classic()+
  geom_point(size=1,alpha=1)
p1=p+geom_smooth()
p2=p+geom_smooth(method = "lm", formula = y ~ poly(x,2))
p3=p+geom_smooth(method="lm")
p
p2
p3
p_Corynebacteriaceae_growth <- p2

p=ggplot(all.growth.sub,aes(x=log(Dietziaceae),y=g_rate))+
  labs(x="Log abundance Dietziaceae ", y="Relative weight change") +
theme_classic()+
  geom_point(size=1,alpha=1)
p1=p+geom_smooth()
```

```
p2=p+geom_smooth(method = "lm", formula = y ~ poly(x,2))
p3=p+geom_smooth(method="lm")
p
p2

p_Dietziaceae_growth <- p2


if(!require(patchwork))install.packages("patchwork")
library(patchwork)


p_growth<-
(p_shannon_growth+p_Halomonadaceae_growth+p_Streptococcaceae_growth)/
  (p_Corynebacteriaceae_growth+p_Dietziaceae_growth)
p_growth


ggsave(p_growth, file="growth_abundance_control_stage.pdf",
       width=20, height=10)
```
