# Supplementary material for: Establishment of Gut Microbiome During Early Life and Its Relationship With Growth in Endangered Crested Ibis (Nipponia nippon)
Source: Front Microbiol. 2021 Aug 9;12:723682. doi: 10.3389/fmicb.2021.723682 (PMC8382091; doi:10.3389/fmicb.2021.723682)
Supplement: Supplementary file 12 [file Data_Sheet_2.PDF]

```
# This script is for differential analysis between two adjacent time points

# clean environment variables

rm(list = ls())
options(stringsAsFactors = F)


#  raw reads count of each OTU in each sample. Mean abundance >=0.01%
filter_count <- read.table("count_8OTU.txt", row.names= 1,  header=T,
sep="\t")#get the count matrix

# Design of experiment
metadata = read.table("metadata.txt", header=T, row.names= 1, sep="\t")

#rearrange the column of filter_count according to the row names of
metadata
filter_count = filter_count[, rownames(metadata)]


#assign the variable to another name
exprSet <- filter_count
dim(exprSet)
exprSet[1:6,1:6]

#define variable for individuals and ages
#variable including all the ages
group_list <- factor(metadata$Day2,levels=c("d1", "d3", "d6", "d9",
                                            "d12", "d15","d18", "d21",
                                            "d24", "d27","d30", "d37",
                                            "d44"))
#variable including all the individuals
individual_list <- as.factor(metadata$Individual)

#check the data
data.frame(Sampe=rownames(metadata), individual_list,group_list)

# install and load edgeR package
if(!require(edgeR))install.packages("edgeR")
library(edgeR)

# the design matrix is formed from an  additive model for paired designs.
# we are interested in the average effect of the age over a population of
chicks.
design <- model.matrix(~0+individual_list+group_list)
rownames(design) <- colnames(exprSet)
colnames(design) #group_listd1(day1) as a control
# This creates a design matrix with 26 columns: 14 for the individuals and
12 more for the differences between ages.

# construct DGEList object
DEG <- DGEList(counts=exprSet)

# check the library size
DEG$samples
DEG$samples$lib.size <- colSums(DEG$counts)
DEG$samples
```

```
#calculate the overall count for each sample
library(dplyr)
apply(exprSet,2,sum)
# This is just for checking data.

# normalization
DEG <- calcNormFactors(DEG)


# estimate the dispersion
DEG <- estimateDisp(DEG,design, robutst=T)

# fit glms model
fit <- glmFit(DEG, design)

# conduct likelihood ratio tests for two closest adjacent time points.

#to detect ASVs that are differentially enriched/depleted in day3 vs day1

lrt <- glmLRT(fit,
              contrast=c(0,0,0,0,0,0,0,0,0,0,0,0,0,0,1,0,0,0,0,0,0,0,0,0,0,0)
)


#unannotated the following code for interested comparison
#to detect ASVs that are differentially enriched in day6 vs day3
# lrt <- glmLRT(fit,
#
contrast=c(0,0,0,0,0,0,0,0,0,0,0,0,0,0,-1,1,0,0,0,0,0,0,0,0,0,0)
# )

#to detect ASVs that are differentially enriched in day9 vs day6
# lrt <- glmLRT(fit,
#
contrast=c(0,0,0,0,0,0,0,0,0,0,0,0,0,0,0,-1,1,0,0,0,0,0,0,0,0,0)
# )

#to detect ASVs that are differentially enriched in day12 vs day9
# lrt <- glmLRT(fit,
#
contrast=c(0,0,0,0,0,0,0,0,0,0,0,0,0,0,0,0,-1,1,0,0,0,0,0,0,0,0)
# )

#to detect ASVs that are differentially enriched in day15 vs day12
# lrt <- glmLRT(fit,
#
contrast=c(0,0,0,0,0,0,0,0,0,0,0,0,0,0,0,0,0,-1,1,0,0,0,0,0,0,0)
# )

#to detect ASVs that are differentially enriched in day18 vs day15
# lrt <- glmLRT(fit,
#
contrast=c(0,0,0,0,0,0,0,0,0,0,0,0,0,0,0,0,0,0,-1,1,0,0,0,0,0,0)
# )

#to detect ASVs that are differentially enriched in day21 vs day18
# lrt <- glmLRT(fit,
```

```
#
contrast=c(0,0,0,0,0,0,0,0,0,0,0,0,0,0,0,0,0,0,0,-1,1,0,0,0,0,0)
# )

#to detect ASVs that are differentially enriched in day24 vs day21
# lrt <- glmLRT(fit,
#
contrast=c(0,0,0,0,0,0,0,0,0,0,0,0,0,0,0,0,0,0,0,-1,1,0,0,0,0,0)
# )



# #to detect ASVs that are differentially enriched in day27 vs day24
# lrt <- glmLRT(fit,
#
contrast=c(0,0,0,0,0,0,0,0,0,0,0,0,0,0,0,0,0,0,0,0,0,-1,1,0,0,0)
# )

# #to detect ASVs that are differentially enriched in day30 vs day27
# lrt <- glmLRT(fit,
#
contrast=c(0,0,0,0,0,0,0,0,0,0,0,0,0,0,0,0,0,0,0,0,0,0,-1,1,0,0)
# )

# #to detect ASVs that are differentially enriched in day30 vs day27
# lrt <- glmLRT(fit,
#
contrast=c(0,0,0,0,0,0,0,0,0,0,0,0,0,0,0,0,0,0,0,0,0,0,0,-1,1,0)
# )

# #to detect ASVs that are differentially enriched in day37 vs day30
# lrt <- glmLRT(fit,
#
contrast=c(0,0,0,0,0,0,0,0,0,0,0,0,0,0,0,0,0,0,0,0,0,0,0,0,-1,1)
# )

#to detect ASVs that are differentially enriched in day24 vs day21
# lrt <- glmLRT(fit,
#
contrast=c(0,0,0,0,0,0,0,0,0,0,0,0,0,0,0,0,0,0,0,-1,1,0,0,0,0,0)
# )


#show the top ASVs
topTags(lrt)

#filtering the results based on logFC and FDR
DEG_edgeR <- as.data.frame(topTags(lrt,n=nrow(DEG)))

fc_cutoff <- 2
fdr <- 0.05
DEG_edgeR$regulated <- "normal"

loc_up <- intersect(which(DEG_edgeR$logFC>log2(fc_cutoff)),
which(DEG_edgeR$FDR<fdr))

loc_down <- intersect(which(DEG_edgeR$logFC< (-
log2(fc_cutoff))),which(DEG_edgeR$FDR<fdr))
```

```
DEG_edgeR$regulated[loc_up] <- "up"
DEG_edgeR$regulated[loc_down] <- "down"

#select the significant differential ASVs
deg <- DEG_edgeR[DEG_edgeR$regulated!="normal",]
dim(deg)
deg
table(deg$regulated)

#saving results
# write.table(deg,"d3-d1-210706.txt", sep="\t")
```
